# On the movement of the honeybee queen in the hive

**DOI:** 10.1038/s41598-025-07093-4

**Published:** 2025-07-01

**Authors:** Jan Blaha, Martin Stefanec, Jiří Janota, Daniel Nicolas Hofstadler, Tomáš Rouček, Jiří Ulrich, Laurenz Alexander Fedotoff, George Broughton, Tomáš Vintr, Farshad Arvin, Thomas Schmickl, Tomáš Krajník

**Affiliations:** 1 Faculty of Electrical Engineering, Czech Technical University, Prague, Czechia; 2 Artificial Life Lab, Institute of Biology, University of Graz, Graz, Austria; 3 Department of Computer Science, Durham University, Durham, United Kingdom

**Keywords:** Computational biology and bioinformatics, Statistics, Animal behaviour

## Abstract

A honeybee colony is a complex and dynamic system that emerges out of the interactions of thousands of individuals within a seemingly chaotic and heterogeneous environment. At the figurative core of this system is the honeybee queen, responsible for the growth and reproduction of the eusocial superorganism. In this study, we examine the interaction between the queen and her surrounding environment by analyzing her movement patterns using mathematical models and computational approaches. We employed a visual tracking system to observe three queens of *Apis mellifera* within their colonies over a three-week period and analyzed sets of quality tracklets to provide observational evidence regarding the queens’ motion-related decision-making. Contrary to expectations, we found that the queen’s short-term motion characteristics—such as speed and turning—were remarkably invariant across distinct hive regions, suggesting a lack of direct environmental modulation at short timescales. Yet, long-term patterns showed structured and strategic behavior. Inter-stop distances followed a power-law distribution, and queens repeatedly revisited specific spatial zones over multi-day timescales. These results indicate a dual-scale movement strategy that is not captured by standard random walk models, highlighting internal state or memory-based navigation. Our findings suggest that queen movement is shaped by temporally layered processes that may support brood nest stability, efficient egg-laying, and colony cohesion.

## Introduction

Honeybees (*Apis mellifera* L.) are an integral part of many ecosystems^1^, maintaining biodiversity^2^ and increasing agricultural productivity^3^, primarily through their contribution to pollination^4^. A typical honeybee colony consists of tens of thousands of individuals that function as a highly organized and efficient unit^5^, with many aspects of colony regulation arising from the collective behavior of its members^6–8^. Among all the members of the honeybee colony, a single central individual, the queen bee, is instrumental in maintaining the colony’s cohesion, growth, and reproduction^9^.

This centralizes the responsibility for the colony’s growth and reproduction in the queen bee alone. To maximize efficiency, the queen must carefully balance exploration and exploitation to ensure the worker population reaches its optimal size. Such a need to choose between refining current strategies (exploitation) and discovering new opportunities (exploration) represents a classic trade-off in biology, the exploration-exploitation dilemma. Her capacity to lay thousands of eggs daily hinges on several factors: the availability of essential nutrients, the presence of empty, prepared cells, and her ability to locate these cells within the hive. The queen can show “exploitative” motion patterns, allowing her to lay eggs in spatially compact areas, maximizing her egg-laying efficiency and contributing to the colony’s energy management. Brood cells are not distributed uniformly throughout the hive but occur clustered and densely packed at the center of the hive, forming the “brood nest”. Parts of the brood nest area in a hive may be on both faces of a comb or, in larger hives, may even span several combs, often not directly connected, requiring the queen to navigate between them. Since the development of the honeybee brood requires substantial amounts of energy and accurately controlled thermal conditions^10^, the dense clustering, coupled with thermally insulating cells around the outer edge of the brood nest area^11^ provides advantages in heating efficiency. In addition, the high density of brood cells minimizes the walking distance for the queen between egg-laying sites. On the other hand, the queen can display “exploration” behavior, actively searching the hive to find clusters of empty cells (freshly prepared by worker bees for her to lay eggs in) and dispersing pheromones that regulate key colony behaviors^12^. In summary, the queen must balance exploration to locate suitable egg-laying sites and distribute pheromones with the exploitation of the brood nest area to maximize her egg-laying efficiency.

Before oviposition, the queen evaluates the suitability of a cell by tilting her head toward it. If she finds a cell suitable, she advances slightly, inserts her abdomen into the cell, and deposits an egg. This process, which lasts several seconds, momentarily interrupts her movement, resulting in a short-stop in her movement. Many research efforts have explored the emergence of the brood nest through modeling approaches ^13–16^. For these models, the movement behavior of the queen is simply assumed to be a random walk. Understanding the queen’s movement can shed light on the inner workings of honeybee colonies and inform an ongoing scientific debate, as aspects of movement affect the formation of structures such as the brood nest area over time.

As the queen’s behavior during egg-laying involves searching for suitable cells, we expect to find typical properties of the searching behavior of insects, which depends on the sensory input the animal perceives, intrinsic motivations, and external environmental factors^17^. In the case of honeybees, such external factors could be comb layout, geometry, gravity, temperature gradients on the comb, or interaction with other bees. Related studies have shown that decision-making processes can be reflected in movement patterns, either influenced by environmental or internal factors^18^. Finding differences in the short-term movement characteristics between different areas should thus allow us to derive a deeper understanding of the decision-making processes of the animal. An animal’s movement behavior can also be substantially influenced by endogenous cycles, such as circadian rhythms^19^. If such temporal patterning is prominent, simple models like random walks, which often neglect these dynamics, would likely prove insufficient to capture the full complexity of the observed behavior. In our research, we formulated several questions. We wanted to investigate whether the exploration-exploitation trade-off is discernible in the queen’s motion between the different hive areas with respect to their distinct functions—brood nest and storage (non-brood nest) areas. Reflecting the queen’s optimization strategies for colony growth, her movement behavior in the brood nest areas, where egg-laying activities are concentrated, should differ from her movements in the storage areas. We also aim to determine whether the macroscopic movement patterns of the honeybee queen can be derived from microscopic movement patterns, thereby linking microscopic measurements to macroscopic outcomes.

To systematically investigate these questions, we used methods adopted from the field of movement ecology^20^, which aims to understand the factors and mechanisms that influence the movement patterns of living organisms. Such movement patterns have been extensively studied in larger animals such as birds^21^, marine predators^22^, and mammals^23^, providing valuable insights into animal ecology^24^ and the decision-making processes^25^, the evolutionary aspects^26^, or the impact of human activities on habitat use^27,28^. The development of modern tracking technology, like radar tracking^29^, GPS^30^ or eDNA^31^, allowed for great advances in the field, but brings new questions as well^32^. From trajectory data, descriptive models have been constructed, which model key characteristics of the animals’ movement behavior^33,34^. These models describe the trajectory polylines as trails of a random walk process; in some cases, this seems to be an emergent property^35^, in others, there is evidence that the generative process itself is the respective random walk^36^. Models built on random walk theory were used to describe, for example, the motion of higher-order vertebrates like birds^37^, jackals^38^ even humans^39^ as well as marine predators^40^, mussels^41^ and insects like fruitflies^42^, butterflies^43^, honeybees^44^ or ants^45^.

We present a study of a 22-day long-term dataset we collected using a marker-based visual tracking system of three queens in their hives, with over 34,000 tracklets of the queen trajectory. These tracklets provide us with short-term observations of the movement behavior of an individual. Previous work on insect trajectories worked with much longer segments, e.g., hundreds of meters^29^, but these works tracked animals in open environments. In contrast, our work focuses on an animal in the confined space of the hive, where the longest possible motion on a straight line would be 47 cm long. Therefore, our observations are representative for the scale of the animal movement.

We investigate fundamental properties of the queen’s behavior, analyzing activity levels and their spatial dynamics. To distinguish between intrinsic movement decisions and extrinsic influences on queen movement, we further fit common random walk models to the empirical tracking data and use simulation to measure their ensemble properties. To investigate the queen’s movement patterns, we employed metrics such as diffusivity, coverage, and first-passage time to measure the explorative aspects of the queen’s motion patterns, and metrics like self-crossing rates and fractal dimension as a measurement of the explorative features of the queens’ motions patterns. We applied these metrics to both empirical trajectories and those generated by our simulations to identify the model that best replicates the observed spatial dynamics. This comparative analysis serves two purposes. It allows us to determine whether a particular model can accurately describe the queen’s movement, and it allows us to draw conclusions on the properties of the movement.

## Methodology

**Fig. 1:**
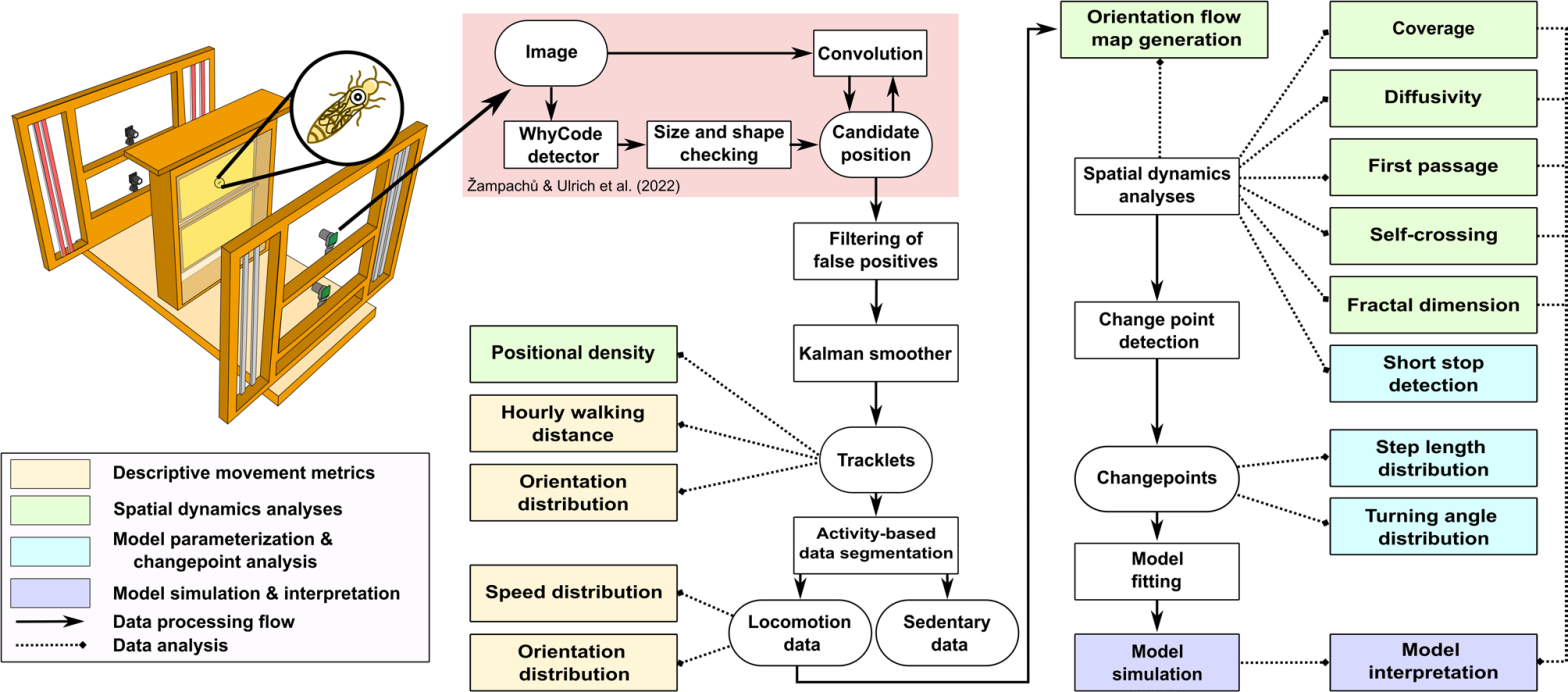
Workflow of Queen Bee Movement Analysis. Upper left illustration: The observation hive, positioned between two panes of glass, was monitored by four cameras that captured all activity across two honeycombs (the lighting setup is illustrational, see Supplementary Methods 1: “Dataset Collection and Processing” for details). Data analysis begins with the WhyComb detector system identifying the tagged queen in the images, followed by size and shape verification to generate candidate positions. These positions undergo convolutional network verification, which leads to candidate refinement by filtering out false positives and generating validated tracklets. A Kalman smoother refines these tracklets for descriptive movement analysis, which includes positional density and hourly walking distance. The data is then segmented into mobile and stationary phases, with the former analyzed for velocity and orientation distributions. Spatial dynamics, including orientation flow map, coverage, diffusivity, first-passage, self-crossing, and fractal dimension, are computed along with short-stop detection. Change point detection facilitates the analysis of step length and turning angle distributions, upon which models are fitted for simulation and comparison with empirical spatial dynamics data.

Figure 1 depicts the workflow of the full data analysis approach, showing the processes from data collection, data refinement, descriptive motion analysis, and spatial dynamics analysis up to model simulation and interpretation. Our observation spans three honeybee colonies over a period of 22 days (from August 25, 2022 to September 15, 2022, totaling 528 hours), which covers a whole brood cycle of worker bees (21 days). To the best of our knowledge, no dataset of this extent focusing on the movement of a honeybee queen has been previously published.

### Animals observed in this study

This study used three honeybee colonies (*Apis mellifera carnica*, Pollmann) housed in three standard observation hives, each containing two combs. It is an observational study in its entirety. The bees were allowed to leave the hive *ad libitum* and forage in the surrounding environment, with each observation hive containing approximately 3,000 to 5,000 individual bees. There were no specific criteria for inclusion or exclusion of honeybee colonies or honeybee queens in this study.

The colonies were commercially bred by the Styrian Beekeeping Center in Graz, Austria, and maintained by professional beekeepers at the University of Graz. The management of the colonies followed strict animal welfare guidelines in accordance with the Austrian Animal Experiments Act (TVG 2012, 1. Abs., §1) and the standards of the Ethics Committee of the University of Graz.

### Data collection

Each observation hive was equipped with two combs to facilitate comb-type dependent segmentation, allowing for the differentiation of movement behavior between brood and storage combs within the dataset. For each comb side, one 4K resolution camera was mounted, making a total of four cameras per hive. Camera ArduCam HQ-IMX477 4056x3040@10 Hz with switchable IR filter (in our case always off) with a CS2008ZM05A F/1.2 lens was used. Each camera is mounted 47 cm from the comb plane giving  1,720 px per cell or  $63\,\hbox {px/mm}^{2}$.

The scenes were illuminated in near-infrared light to minimize disturbance to the colonies and queens. For illumination, 8 LED light bulbs 9 W@850 nm were used on each side of the hive pointing to the middle of the comb. See Supplementary Methods 1: “Dataset Collection and Processing” for more details on the system setup.

Over time, the bees produced a lot of debris and other biological material that stuck to the glass; this severely limited any automated observations. In terms of disturbance, our hives are maintained with periodic clean glass replacement, and one of these glass replacement events occurred during the period of recording data for experiments in this paper.

As noted in the previous Section, many different technologies have been used for the study of animal movement and behavior based on different modalities. For our work on insects, we make use of the fact that the area where the queen can move is restricted; therefore, it can be completely covered by a camera-based visual system. Historically, marker-based systems were used to observe honeybees both manually, e.g.^46^, and automatically, e.g.^47^. There have also been markerless approaches, such as tracking of the worker bees purely based on visual data^48^, those however so far, only provide short-term individual identification. In this study, we used a previously introduced marker-based tracking system called “WhyComb”^49^ in order to achieve robust and reliable tracking of the queen bee throughout an entire brood cycle.

WhyComb extends the established high-precision robotic positioning system WhyCon^50^ and is adapted to address task-specific challenges such as cluttered hive environments and frequent marker obstructions by crawling bees. For a detailed description of the whole system data collection, see Supplementary Methods 1: “Dataset Collection and Processing”. The detection system, however, still produced a substantial number of false positive detections, mainly due to clutter in the scene. This required post-processing of the data, for which an algorithm to classify false positive detections was developed. In the used configuration, the camera’s frame rate was about 10 Hz, setting the maximal attainable rate of candidate detections. The filtered detections were then smoothed out using a Kalman smoother to remove the noise in the marker positioning and to account for the queen’s non-walking movement (for example, in her roll axis), see Supplementary Methods 2: “Cleaning Data” for details.

Subsequently, from our raw dataset, we extracted intervals of quality observations—temporally dense, continuous segments of each queen’s trajectory, termed “tracklets”. These tracklets were generated independently for each of the four cameras due to the limitations of precision when transforming coordinate systems across different cameras and individual combs. In total, 34,384 tracklets of a queen’s trajectory were extracted, see Supplementary Methods 3: “Tracklets” for details on the production of tracklets from raw data. Of those where the queens walked, 47 % were over 5 cm long with average length of 17.95 ± 16.35 cm, these tracklets covered 89 % of recorded distance. The longest tracklet reached 2.18 m. In total, in hive 0, we recorded 821.84 m of tracks, in hive 1 it was 470.93 m and in hive 2 1146.96 m.

Apart from false detections and occlusions by other bees, the queen also spent time traveling in between the comb planes around the hive construction or through tunnels where it was not possible to track her. The tracking success rate was high in hives 0 (80%, 421 hours of tracklets) and 2 (81%, 427 hours of tracklets), with hive 1 being substantially worse (31%, 162 hours of tracklets). Worse observation conditions in hive 1 enabled us to assess how our methodology and its conclusions were affected by varying success rates of the tracking.

Finally, based on the speed information, we segmented the tracklets into the walking and resting behaviors. Using only the trajectory data, the resting behavior is hardly definable as there are multitudes of reasons why the queen could stop, such as cell inspection, oviposition, feeding, or interactions with surrounding bees. We, therefore, also differentiate between a longer resting period and a momentary stop, getting an additional activity mode, which we call a short-stop. Out of the variety of reasons for the queen to stop for a short time, like cell inspection or interaction with workers, egglaying is particularly interesting for us. While equating short-stops with egglaying events is speculative, in^51^ authors show about 50 to 70 % correlation for similarly defined events they call “candidate egglayings” events. See Supplementary Methods 4: “Activity Segmentation” for technical details on the activity classification.

To provide a detailed description of the queens’ motion, we began by examining descriptive movement metrics that encompassed spatial and temporal properties, such as the queens’ hourly walking distance, positioning on the combs, and distributions of orientation and speed. Where appropriate, we look at the data separating the lower and upper combs to study the differences. The front and back sides of all the combs were considered together—reflecting the fact that they usually serve the same purpose—with the back side flipped horizontally to match the view from the front side.

### Spatial dynamics analyses

According to literature^33^, polylines can effectively represent animal movement. The generative process can then be modeled as a continuous random process in $\textbf{R}^2$, characterized by a step length distribution *p*(*l*) and a turn angle distribution $p(\phi )$. In contrast to more traditional formulations of random walk models in mathematics, these models work with orientation change between steps, not the distribution of general orientation, and are sometimes referred to as “step-and-turn models”. Despite its simplicity, this formulation serves as a basis for constructing a rich class of models of motion by varying the governing laws.

There are two principal distinctions between these models that have direct biological implications—the presence of isotropy, which refers to orientation indifference, and whether step lengths follow an exponentially or polynomially decaying distribution. When the process is not isotropic, it has correlated orientations of successive steps, which defines correlated walks. These specifically exhibit directional persistence over a longer time; the agent is more likely to move in the direction it is already moving in and reach farther. To what extent this happens is affected by the concentration of turning angles. The decay of the distribution—or tail—is then important because the probability of large steps is the deciding factor when it comes to the motion characteristics. For exponentially decaying distributions, the characteristics converge to those of a Brownian Walk (BW), where *p*(*l*) follows a $\text {Rayleigh}_2(l)$ distribution with a tail $p(l) \sim e^{-l^2}$. Conversely, polynomially decaying distributions, like the Lévy Walk (LW), governed by one of the Lévy alpha-stable distributions with a tail $p(l) \sim l^{-\mu }$, yield a wider range of motion patterns depending on the Lévy exponent $\mu \in (1, 3]$^52^. In particular, a $\mu$ value of 3 leads to a normal distribution, values less than 3 result in the absence of the second moment, and $\mu$ values less than 2 result in the absence of the first moment. Unlike the ones with exponential tails, walks governed by distributions with polynomial tails can exhibit directional persistence without correlated turning angles.

We used five key metrics to categorize the patterns arising from the queen’s movement based on their relevance to either exploratory or exploitative behavior. Three of those were chosen to analyze the queen’s exploration activities, specifically an analysis of the diffusivity of her walking behavior, a first passage analysis, and an assessment of the area covered relative to the distance traveled. To assess exploitation aspects, we analyzed self-crossing paths and the fractal dimension of the tracklets. We fitted all properties with simple mathematical models appropriate for the given property on the range of walked distance of [0,25] cm, as we wanted to have results comparable across all hives and separate most of the confinement effect. The fractal dimension then provides insight into the local complexity of the movement. As we wanted to inspect the behavior on the scale of tens of centimeters, we did not include tracklets of less than 5 cm as these did not bring more information despite their volume.

### Ensemble walk properties

For a random walk process $(X_t)_{t>0}$, the *diffusivity* $\langle X_t^2 \rangle$ is the mean-squared displacement of the walker over time, providing insight into the walker’s speed of movement through its environment (see Supplementary Methods 5: “Modes of Diffusivity”). The asymptotic behavior of the diffusivity indicates the efficiency with which the walker explores its environment. The superdiffusive mode of the Lévy walk is one of the arguments for its foraging optimality^53^. Diffusivity is a cumulative function starting at zero, characterized mainly by its order, so we modeled the diffusivity as a function $f(x) = a x^b$.

Aside from the speed of diffusion of a theoretical ensemble of walkers, the authors argue that for a foraging animal, a more interesting property is the *mean-first-passage-time* (MFP)^18,37^. MFP quantifies the expected time required for an animal to reach a given distance from its starting point for the first time, it quantifies how fast an animal can get far away from its original position. It is defined as a function $\text {MFP}(D) =\mathbb {E} \big [ \mathop {\textrm{argmin}}\limits _t \{X_t; \text {dist}(X_0,X_t) > D\}\big ]$, where *D* is the displacement threshold and *t* is time. We modeled it as a function $f(x) = a x^b$.

The *areal coverage* was quantified as the proportion of a 1 mm grid superimposed on the accessible movement area effectively covered by the queen, modeled as a circle of 2. 5 cm radius. This metric aims to quantify the queen’s perceptive range, suggesting that she does not need to physically cover the complete comb to exert her influence on a given location within the hive. Areal coverage is also a cumulative function starting at zero, but since we consider the queen’s own perceptive range to be a circle, we get a common intercept parameter, so we modeled it as a function $f(x) = a x^b + c$. An alternative perspective is given by inspecting the *self-crossing* of queen trajectories, as how often the queen’s path crosses itself over time is tied to repeated visits to the same locations. This metric provides insight into the redundancy of the queen’s patrol patterns and potential areas of concentrated pheromone deposition. To compute it, we counted intersections of the tracklet with itself along the observed trajectory. However, to filter loops due to small jitter in the position, we imposed a minimal walked distance of 2.5 cm in the loop and between two consecutive crossings, corresponding to our considered perceptive range of the queen. Self-crossing was also modeled as a function $f(x) = a x^b + c$.

In the literature on random walks, it is usual to assume the constant speed of the walker; however, the queen does not meet this assumption. Anywhere we would use time, we need to renormalize it by her momentary speed to make the results comparable and interpretable. This effectively means using her walked distance in the place of time.

### Fractal dimension

Following the literature^38^, we examined the *fractal dimension* of the queen’s movement paths. This is a particular measure of the geometric complexity of her trails, which allows us to quantify her “wiggliness” on a scale from a straight line with a dimension of one to a plane with a dimension of two. The random walks we considered produce self-similar fractal patterns and lie in between these two extremes. While BW theoretically achieves full surface coverage with a fractal dimension of two, the queen’s movements exhibit varying degrees of complexity, indicative of her strategic foraging and pheromone distribution behaviors. To analyze the fractal characteristics of the queen bee’s motion, we used the commonly known box-counting algorithm, following literature^54^ (see Supplementary Methods 6: “Estimating Fractal Dimensions” for details).

### Changepoint detection and movement models

Insect movement modeling is fundamentally based on “step-turn” models. Analysis of raw trajectory data requires preliminary identification of points of changes in the trajectory direction, called changepoints, to segment the path into discrete steps. To characterize the step length distribution, we chose a geometrical method based on the ratio of eigenvalues^55^ that is commonly used to identify corners in point data. We compute a covariance matrix from a small temporal window on her trajectory and then search for local maxima of the ratio of eigenvalues, indicating low collinearity of consecutive points. Details are given in Supplementary Methods 7: “Changepoint Detection”. While not substituting changepoints, for certain analyses, short-stops were used as points of interest on the queen’s trajectory, as described in detail above and in Supplementary Methods 4: “Activity Segmentation”.

Different models were then fitted to the step and angle distributions. For turning angles, we considered the uniform and von Mises distributions; for step length, we considered Squared Exponential, Exponential, Pareto, Truncated Pareto, and Exponentially Truncated Pareto distributions. Care must be taken to account for the restricted area of the comb, which affects especially the power-law distributions with potentially infinite second moments. We deal with the confinement and the behavior at the edges by truncating any step distribution so that the walker, given its position and orientation, cannot exit the comb area.

### Model testing

To evaluate the accuracy of our models in reflecting empirical data, we take a two-fold evaluation approach. First, we examine the statistical fit of the distributions to the empirical data, followed by an examination of the ensemble behavior expected from the models. To understand the ensemble behavior, we cannot rely on purely theoretical results since our case violates the assumptions of the models by having a confined area, an agent of non-constant speed, and including the probabilities of small steps. Therefore, we resorted to the simulation of walkers using the fitted parameters.

The data for fitting the models was taken as a sample of the whole dataset. The first day was taken completely, and one different hour was taken from each following day. This way, the training data covered temporal detail as well as longer changes over the observation period.

To fit the von Mises distribution for turning angles, we used a numerical approximation of the maximum-likelihood estimate (MLE)^56,57^. The approach to analyzing step lengths is not so simple. Discussions within the scientific community^40,58–61^ have led to the preference for the use of MLE for parameter estimation and the Akaike Information Criterion (AIC) for model comparison. Unlike analytical solutions in the literature, our models had to be fitted using numerical optimization methods due to the truncations caused by confinement.

In order to perform MLE fitting of the parameters, one also needs to know the beginning of the distribution’s tail $l_{min}$, as that is to be fitted. We adopt a solution that has been proposed in^62,63^ for the fitting of universal power-laws. The authors propose trying to fit the models for a range of $l_{min}$ values and taking the one with the smallest Kolmogorov-Smirnov distance between the empirical data distribution and the fitted model. For details on the model fitting, see Supplementary Methods 8: “Fitting Random Walk Models”; details on individual models are then available in Supplementary Methods 9: “Considered Model”.

The simulation of the models did not follow straightforwardly from their definitions because of two issues. First, the distribution of steps was only fitted for its tail. We supplied the body of the distribution by a trivial model approaching 0 from the right side—the half-truncated normal distribution on the interval $[0, l_{min}]$, with location set to $l_{min}$, and its mass corresponding to the quantile of the empirical step distribution at $l_{min}$. Its scale was fitted to the data using MLE. The second issue was the unbalanced length of empirical tracklets; therefore, the simulated trails were generated for each empirical tracklet with the corresponding length. Individual models were then implemented by uniformly sampling the starting point and subsequently independently sampling the angle and length in each step. For details on the model simulation, see Supplementary Methods 10: “Simulation of Random Walk Models”.

## Results

### Descriptive movement metrics

In observation hives 0 and 2, the brood nest was located on the lower comb. In hive 1, the brood nest area was located on the upper comb for the majority of the observation period. However, during the end of the observation period, the queen began laying eggs on the lower comb, establishing a new brood nest area.

**Fig. 2:**
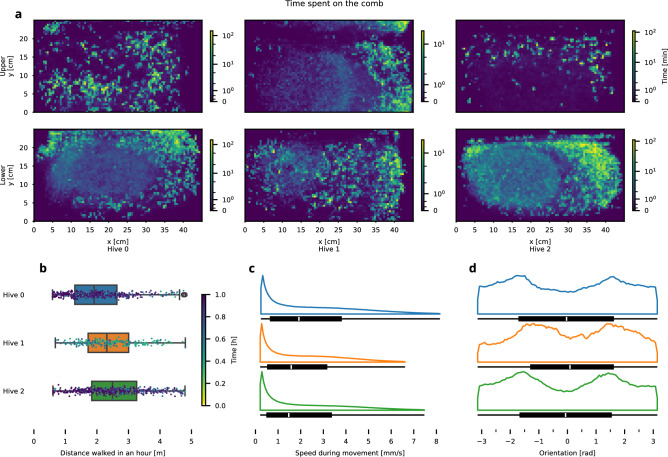
Summary of queen movement observations. Panel (**a**) shows the total recorded time of the queen on the combs as a heatmap on a 0.5 cm grid (**b**) shows the activity levels by estimating the average distance the queen walks in hourly aggregates, renormalized by the observed time, where at least 30% of the data was captured. Together with the total distance walked, the distribution of the queen’s walking speed (speed with probability of walking higher than 0.5, see Supplementary Methods 4: “Activity Segmentation”) can be obtained, with high outliers filtered by taking the 99th percentile (**c**). Panel (**d**) shows the distribution of queens’ orientation on the comb while walking.

Our analysis of queen movement metrics shows that the three queens have traveled distances from 0.5 to 4.5 m in an hour, with approximately a 30% difference among them, see Fig. 2**b**. The queens exhibited similar traveled distances, with median values ranging from about 1.9 m for queen 0 to about 2.5 m for queen 2. The distribution of velocity across the hives was very similar, with an average velocity of about 2 mm/s and slightly below. High speeds were relatively uncommon; queens predominantly moved at speeds of up to 6 mm/s, as shown in Fig. 2**c**.

Figure 2**d** illustrates the orientation distribution of the queens while walking. Due to the orientation being estimated from motion, the data points were included only when changes in position were larger than 0.5 mm to filter unstable estimates caused by jitter in detections. It is noteworthy that the distribution is bimodal.

### Spatial dynamics analyses

**Fig. 3:**
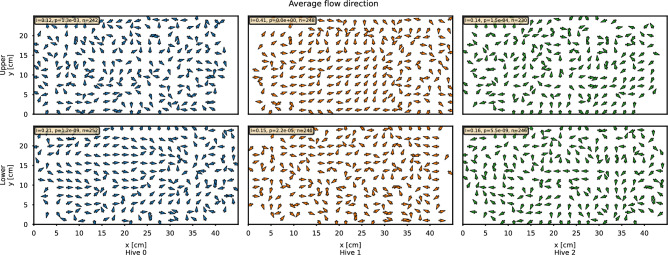
Spatial patterns in queen movement in longer horizons. The figure shows the average direction of movement per cell in a coarser 2 cm grid. For each comb, we also give the generalized Moran’s I, which measures the spatial autocorrelation of directions between neighboring cells and the corresponding p-value of the hypothesis that there is no particular arrangement in the emerging flows.

Positional density heatmaps of Fig. 2**a** indicate different preferences exhibited by the queens in different areas of the hive. The brood nest area emerges when looking at the spatial coverage on the lower comb in hive 0 and hive 2, similar, less pronounced, pattern can be seen on both combs of hive 1, which is in line with the new brood nest emergence we observed. Movement analyses reveal distinct patterns of directional preference across all combs when looking at the average direction^56^, see Fig. 3. We computed the generalized Moran’s I statistic^64^—which is a particular measure of spatial autocorrelation—for different settings of the neighborhood function. For immediate neighbors, Moran’s I values (given in Fig. 3) ranged from 0.1 to 0.4, indicating a positive correlation in the average direction between adjacent cells. Using the permutation-based method, we determined p-values to test the hypothesis that the data on queens exhibit no spatial arrangement (orderliness) in their movement. The least significant result was observed in the upper comb of hive 0, with a p-value of $p=8.5 \times 10^{-4}$; we therefore conclude the presence of pronounced spatial patterns in the flows. For details on the definitions and Moran’s plot, including results on different neighborhood variants, see Supplementary Methods 11: “Moran’s I”.

**Fig. 4:**
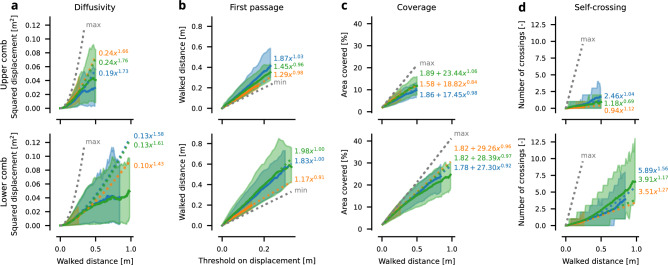
Empirical observations of spatial dynamics properties. We show the mean and interdecile range of the observations for each hive (following the color scheme: blue hive 0, orange hive 1, and green hive 2). Values are calculated from individual tracklets of varying lengths. The range of values is clipped so that we have at least 30 observations to estimate the mean. To avoid the presence of confinement effects and to make them comparable between hives and combs, the fits are computed from data up to 0.25 m and shown as dashed lines. Each property further has a theoretical limit, either higher or lower, shown as a “max” or “min” curve.

### Diffusivity

The values of the exponent *b*, specifying the order, are in the range of [1.66, 1.73] for the upper combs and of [1.43, 1.61] for the lower combs and, therefore, for all areas qualitatively very similar, see Fig. 4**a**. This range lies between the theoretical extremes of 1 and 2, with an exponent of 1 corresponding to normal diffusion, while values greater than 1 indicate an anomalous diffusion pattern, described as “superlinear”. The model tends to overestimate the queen observations for larger traversed distances. This discrepancy is attributed to the limited space of the comb, which inherently restricts movement, in contrast to theoretical models that assume unbounded diffusion. The theoretical maximum was calculated as a square of the walked distance, corresponding to the ballistic motion.

### Mean first passage

Our analysis shows that queens 0 and 2 exhibit remarkably similar behavior, with the only notable deviation observed in queen 1, see Fig. 4**c**. Despite these differences, the same simplified model used to analyze diffusivity also very well fits the mean first passage data for all hives ($R^2\approx {1}$). The exponent *b*, between 0.96 and 1.03 for upper combs and between 0.91 and 1.00 for lower combs, matching a consistently linear relationship across all combs and all hives. The value of scaling factor *a* differs more between hives. For hives 0 and 2, *a* approaches 2 from below for both lower and upper comb, suggesting that the queens require, on average, short of twice the distance traveled to achieve a given displacement. For hive 1, this value is a bit lower while still being very similar for the lower (1.17) and the upper (1.29) comb. The theoretical minimum was calculated as the walked distance, i.e. the behavior of the ballistic motion.

### Areal coverage

Coverage showed great consistency across all the combs and hives observed, see Fig. 4**d**. After traversing approximately half a meter, which is approximately the 47 cm diagonal length of the real comb, each queen achieved coverage of approximately 15 % of the area of the comb. A fit of the mean gave very similar results across all hives. In particular, the exponent fitted $b\approx {1}$ for all queens (for the upper comb between 0.84 and 1.06, for the lower comb between 0.92 and 0.97). This indicates that, on average, the queen revisits only a limited number of locations while moving over a distance of 25 cm. The theoretical maximum was computed by sampling a snake-to-side meandric sweep with different starting positions to attain the fastest possible coverage for a given circular perceptive range.

### Self-crossing

The rate at which the queen crosses her own path is low, see Fig. 4**b**. We computed the theoretical maximum crossing rate as a linear function of the walked distance as if the queen would cross her own path every 2.5 cm, which was defined as the minimal distance between crossings. On average, after walking 50 cm, she crosses herself only about 2 times, whereas the theoretical maximum would have been 20 times. The fitted models exhibit substantial differences in both the exponent *b*, which is above 1 due to the confinement effects, and the scaling coefficient *a*. However, the models do not extrapolate well beyond the interval of the training data.

### Fractal dimension

The analysis revealed similar fractal dimensions *D* of queen tracks for hives 0 and 2. Estimates on the lower comb being $D\approx ({1.111 \pm 0.005})$ and $D\approx ({1.125 \pm 0.005})$, while hive 1 had a slightly lower dimension of $D\approx ({1.083 \pm 0.008})$. On the upper comb, the dimensions were consistently lower, for hive 0 $D\approx ({1.076 \pm 0.011})$, for hive 2 $D\approx ({1.072 \pm 0.008})$, and for hive 1 again slightly lower $D\approx ({1.058 \pm 0.002})$. This is likely a result of the generally shorter tracklets on upper combs.

These results suggest a more complex trajectory than a simple straight line, however not much—substantially higher values have been reported for other animals, for example 1.5 for *Canis adustus*^38^. Our results underscore the limitations of using sampled data, which tend to underestimate the actual complexity of the queen’s path. Nevertheless, the results qualitatively correspond to the simulated models, see Supplementary Results 2: “Estimates of the Fractal Dimension”.

### Model parametrization and changepoint analysis

**Fig. 5:**
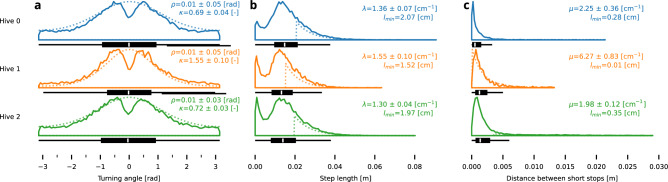
Empirical distributions of turning angles (**a**), steps (**b**), and distance between consecutive short-stops (**c**); and parameters of theoretical models which were judged best by Akaike-weights criteria. The parameters were repeatedly estimated using bootstrapping, we give the means and standard deviations of the estimates. For better readability, we show the distances between short stops clipped to the 95th percentile as with polynomial tails there are extreme values.

The distribution of the turning angles $p(\phi )$—the changes between orientations of consecutive steps—is shown in Fig. 5**a**. The von Mises distribution provided a better fit than a uniform distribution for all hives with mean $\rho \approx 0\,\hbox {rad}$. The concentration parameter $\kappa$ varied, with hives 0 and 2 having similar values of $\kappa \approx {0.7}$, while hive 1 had a higher concentration of $\kappa \approx {1.5}$. The noticeable gap around 0 rad is an artifact of the changepoint detection algorithm, which would not split a perfectly straight line.

The empirical distribution of step lengths *p*(*l*)—the distances between successive changepoints—shows qualitatively similar patterns across all hives examined, as shown in Fig. 5**b**. All hives were best fit by a simple exponential tail. In particular, hives 0 and 2 were fit with similar decay parameters of $\lambda \approx 1.3\,\hbox {cm}^{-1}$. Results for hive 1 showed little higher $\lambda \approx 1.5\,\hbox {cm}^{-1}$. These observations, together with the results of the turning distribution fitting, indicate that the queen’s movement is characterized by an anisotropic, correlated walk with notable directional persistence.

Figure 5**c** shows the distribution of distances between consecutive short-stops by the queen (observed as a part of one tracklet). Across all hives, these short-stop intervals consistently follow power-law distributions. To enhance readability, the plots display data only up to the 95th percentile. This decision is made because the extreme values, characteristic of power-law distributions, extend far beyond the visible range of the plots.

With the power-law being the most supported model, there is variation in the estimated parameters for each hive. Distribution in hive 0 best corresponded with exponent $\mu \approx 2.2\,\hbox {cm}^{-1}$ and hive 2 with exponent $\mu \approx 2\,\hbox {cm}^{-1}$, resembling a Lévy walk. Meanwhile, hive 1 is best described by a power law with a remarkably high exponent of $\mu \approx 6.3\,\hbox {cm}^{-1}$ which would, in limit, collapse into the behavior of exponentially governed motion, but we see, that can be caused by quite low fit for the begining of the tail $l_{min}\approx 0.01\,cm$.

### Simulation results of random walk modeling

**Fig. 6:**
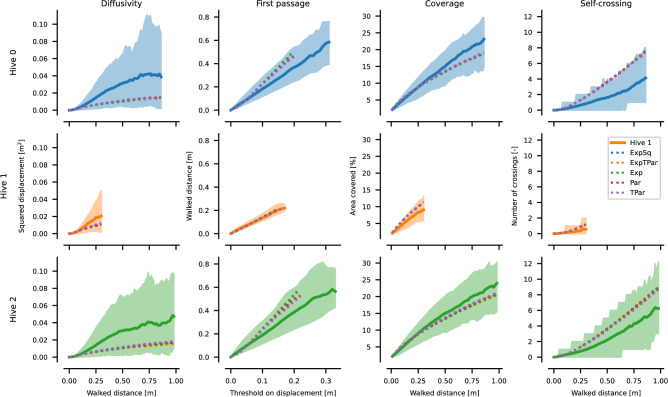
Four basic motion properties of simulated models as compared to the empirical observations. We show the mean and interdecile range of the real observations as solid lines and means for each of the random walk variants dashed. The models all employed von Mises distributions with respective parameters and differed in step distributions—exponentially truncated Pareto (ExpTPar), exponential (Exp), Pareto (Par), truncated Pareto (TPar), and squared exponential (ExpSq).

To compare against the collected data, several different random walk simulations were performed based on the results of model fitting, see Fig. 6. As the turning angles were best fitted by the von Mises distribution, only the results of the correlated walks are presented. While the mean characteristics of the random walks fall within the interdecile range of empirical data, they qualitatively differ from the observed mean in all measurements. Out of all the models, the truncated Pareto approached the empirical data the closest. However, the simulated models exhibited lower diffusivity and coverage, along with higher self-crossing rates and passage times, indicating that even correlated walks tend to display lower directional persistence and revisit the same locations more frequently than the queen. This pattern, combined with lower diffusivity, aligns with the queen’s behavior of leaving an area after exploitation rather than quickly circling back—the models tend to exploit the area more.

### Daily pattern of short-stop activity

Beyond the general characterization of movement metrics and spatial dynamics, we also examined the daily temporal distribution of short-stop events. Given their connection to key behaviors such as egg laying^51^, their distribution over a 24-hour cycle could offer insight into possible diurnal patterning. For this specific analysis, we counted short-stops in all tracklets longer than 5 min and renormalized these counts to a full hour to standardize for varying tracklet durations.

**Fig. 7:**
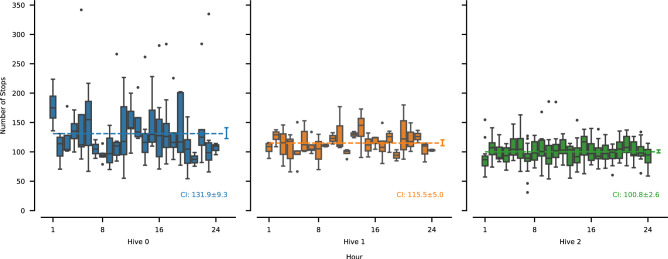
Distribution of short-stop events over the course of the 24-hour period. The number of stops in each tracklet longer than 5 min was renormalized to a full hour. While there is a small notable difference between hives, the number of stops throughout the day does not show any deviation from the uniform distribution ($\chi ^2$ test of good fit for each of the hives, $n=24$, $p=1.0$). The dashed lines show the overall mean and its associated 95%-confidence interval, also given in the figure.

Figure 7 shows the resulting distribution of short-stop events across the day for each hive. The difference in variability between hives is mainly caused by the stability of renormalization and the different average length of tracklets in each hive. Looking at each hive individually, we see no statistically significant deviation in hourly averages from a uniform distribution over the daily 24-hour period ($\chi ^2$ test of goodness of fit for each of the hives, $n=24$, $p=1.0$).

## Discussion

Recent advancements in technology have enabled a deeper understanding of various aspects of the natural world, particularly animal interactions. In this study, we focus on the queen of a honeybee colony, utilizing data previously unattainable through traditional human observations. Our extensive but detailed observations have brought us new insights into the movement behavior of honeybee queens. We can, for instance, assess their median hourly activity (see Fig. 2**b**), which corresponds to a daily movement in the range of 46 to 60 m. Over the three-week observation period, the recorded movement of one of the queens reached a total distance of 1.1 km. The queens exhibited different patterns of activity and rest, with the queens resting between 37 to 61 % of the time.

In the following sections, we investigate how the queen balances brood-focused exploitation with exploratory movement across the hive, and whether her immediate movement patterns differ between brood and storage areas. We analyze spatial statistics such as diffusivity, coverage, and self-crossings, and evaluate how well simple random walk models can account for the observed behaviors. We also consider the potential role of circadian rhythms under constant darkness, and explore how long-term movement patterns might reflect internal states or environmental interactions. Finally, we discuss key limitations of our study, including the number of queens observed and the challenges of long-term behavioral tracking, and outline directions for future research on hive-level behavioral organization.

### Balancing principles

The honeybee queen is faced with a trade-off in her time management strategy. During the colony’s peak growth phase, she can lay hundreds of eggs per day while simultaneously she must “patrol” the hive, distributing pheromones that signal workers about her health and activity state and also regulate worker behavior, heavily influencing the overall colony dynamics. Her oviposition locations cannot be independent, as the brood nest area must be maintained in a compact state to increase the colony’s energy efficiency. Healthy brood development requires a locally precisely controlled temperature environment, which requires a considerable amount of energy expenditure by the workers, making a dispersed brood cell arrangement less favorable.

The typical ellipsoidal brood nest shape found in honeybee hives (a) reflects the (usually rectangular) comb geometry, (b) minimizes walking distances for nurse bees, and (c) ensures heating efficiency for the brood nest that is actively temperature-controlled and heated by the bees. To achieve it, it is required that she frequently inspects cells closer to the area where she has already produced brood. Only if the brood nest is sufficiently compact can nursing and heating expenses be minimized, and rapid brood production can be promoted. Efficient and effective brood production is essential for maximizing colony productivity (honey yield) and winter survival chances (population size). This exploitative localized behavior is in contrast with her coverage of the whole hive, but apart from pheromone distribution and patrolling, exploration of other hive regions also allows her to find suitable new areas for creating additional brood nests, such as on neighboring comb frames in traditional box hives.

As a result of this trade-off situation, we were prompted to question whether the queen’s movement patterns exhibit a dual nature, reflecting strategic behavioral choices between exploration and exploitation in different functional areas of the hive-specifically, the brood nest and storage areas-since specialized movement patterns could lead to increased optimization efficiency. Specifically, we expected to see more exploitative movement decisions in the brood nest, indicative of egg-laying-related activities, and more displacement movement decisions in the storage areas, indicative of exploratory behavior. Such distinct behavioral patterns should be identifiable in our short trajectory segments; therefore these segments would reveal the decision-making processes of the queen. By examining emerging movement characteristics such as diffusivity, coverage, self-crossing, fractal dimension, and first-passage time, we aimed to uncover the underlying processes shaping her immediate movement decisions. In our data, hives 0 and 2 had large brood nests localized strictly on the lower comb, while in hive 1, the brood nest was originally localized on the upper comb, but a new brood nest started emerging during our recording period. Due to this clear separation, we argued for the basic functional areas of brood and storage to correspond to individual combs. Contrary to our expectations, analyses of the chosen statistical characteristics revealed minimal differences across the hives’ functional domains (Fig. 4).

### Immediate decisions and long-term patterns

The immediate decision-making processes could either be based on intrinsic motivation or on extrinsic factors^17^. Our results suggest, however, that the movement behavior of the queen is not primarily driven by immediate decision-making, as the short and medium-term properties of her movement (Fig. 4) are qualitatively comparable between areas with different functions. Nevertheless, the analyses focused on long-term properties reveal distinct differences between brood and storage areas, suggesting either intrinsic behavioral persistence or extrinsic environmental interactions in longer horizons. For example, the queen spends most of her time in the brood nest region, which is consistent with established findings^5,65^, as shown by the positional density in Fig. 2**a**.

A closer look at the movement directions of the queens (Fig. 3) also reveals distinct flow patterns in different hive areas. This view over a longer time period reveals preferred movement paths taken by the queens and repetitive patterns which could, in the future, be studied also using maps of dynamics^66^. The average movement direction is more organized in areas with brood (such as in hive 0 and hive 2 on the lower comb and for most of the observation time on the upper comb of hive 1). As discussed in the previous section, the balancing of different roles would be expected to be associated with different motion behaviors in different areas of the hive. The searching for empty cells would be, for example, affected by varying distribution of brood and storage cells. While in the statistical properties of her movement we did not find any such difference, it is present in generally higher organization of her movement in the brood areas. The fact that we see these patterns pronounced in data averaged over the entire 22-days observational period leads us to believe that it is not caused by any short-term distribution of the cell contents, but rather to be caused by a more persistent external factor or internal. Still even longer observations might be needed for such conclusions.

The analysis of queen movement orientation aggregated overall time and area showed a bimodal distribution with notable peaks at approximately $-90^{\circ }$ and $+90^{\circ }$. A uniform orientation distribution would be expected for an isotropic random walker, like the one following the Brownian motion; suggesting that when walking, the queens more often face either the left or right side of the comb, rather than up or down (see Fig. 2**d**). The total accessible area for the queen in our setup was almost square and consisted of two vertically stacked combs, showcasing that her orientation pattern was not purely a consequence of this spatial arrangement. However, the queens would spend more time on one comb before moving to the other, rather than moving directly between combs. Thus, it is likely the geometry of one comb that influences the emerging orientation distribution, not the square area of the hive, as the queens more often walk toward the left or toward the right of the individual rectangular combs.

These results point to the existence of different behavioral patterns between temporal scales of observation, suggesting that while immediate movement decisions may not vary much across different hive areas, patterns in her behavior emerge over longer periods. While the queen seems very consistent in her immediate decisions, these long-term patterns may indicate some form of (self-)awareness. Over the long term, she repeatedly follows similar paths through the hive, creating a repetitive pattern of movement, even though the immediate decisions appear to be independent of location. We believe that this work might add to a growing body of literature^67^ suggesting that insects also have more complex inner lives than previously thought and may even present some form of consciousness.

### Simple movement models

One of the commonly accepted premises is, that the movement of the queen could be described as a form of a “step-and-turn” random walk. To test this hypothesis, we tried to fit several distributions of step lengths and turning angles (see Fig. 5), which specify variations of common random walk models. To gain more insight, we then tried to qualitatively replicate in simulation the properties found by the analyses of our empirical data. Although the process of fitting such models is consistent with common methods in animal movement analysis, the properties in the collected data differed from the results predicted by our simulations (see Fig. 6).

In our work, we considered several typical models commonly used in similar studies, which correspond to different forms of random walks that are characterized by different statistical properties. Our results show that none of these random walk models successfully produced a movement pattern similar to that observed in the queen. In all three hives, all tested models underestimated the diffusivity compared to the empirical data. For the first passage analysis, all tested models overestimated the walking distance needed at different thresholds of displacement in hive 0 and hive 2. Coverage was underestimated for hive 0 and hive 2 for all models. Collectively, these three metrics indicate less directed, less exploratory movement for all models compared to our empirical measurements. The number of self-crossings was overestimated for all hives in all models, indicating more exploitative movement patterns of the models compared to our empirical data.

This discrepancy highlights a fundamental oversimplification in the standard random walk models, which assume that movement is Markovian (memoryless), homogeneous, and uninformed—fully determined by fixed step and turn distributions without influence from internal states or external stimuli. Such models are inherently limited in capturing behavioral processes that depend on past experiences, internal drives, or environmental feedback, all of which are highly plausible in the context of honeybee queen behavior. For instance, queens may adjust their movement patterns in response to brood distribution, pheromone concentrations, or recent oviposition activity-factors that violate the independence assumptions at the core of these models. Furthermore, these models presume a featureless, isotropic environment, whereas the hive is spatially heterogeneous and dynamically structured. Thermal gradients, chemical signals, and the evolving brood landscape create a rich informational field that likely guides the queen’s actions. The mismatch between model predictions and empirical data thus reflects more than parameter misestimation—it points to a failure of the modeling framework to accommodate context-sensitive, goal-directed, or feedback-driven behavior. Additionally, stochastic models that assume stationarity and locality are poorly suited to capture long-term structure, for models of colony behavior over the whole season a model able to reflext external factors will be neccessary. We interpret these limitations as evidence that queen motion is governed by a complex interplay between internal physiological states and a richly structured environment. Future modeling efforts will need to move beyond naive random walks, incorporating spatial heterogeneity, temporal dynamics, and biologically grounded behavioral rules to more accurately reflect queen decision-making in situ.

### Circadian influences on the queen movement

The temporal organization of behavior in honeybee colonies is well-documented, with worker bees, particularly foragers, exhibiting robust circadian rhythms in activity, entrained by environmental cues like light-dark cycles^19,68^. This might lead to an expectation that the queen, the central reproductive individual, would also display distinct circadian patterns in her in-hive activities. If queen movement and key behaviors like egg-laying or cell inspection were strongly modulated by an endogenous daily rhythm, simple movement models like those tested in our study, which do not account for such temporal periodicity, would inherently be insufficient.

While queens possess an endogenous circadian clock system that can be entrained by light-dark cycles (LD) and influence egg-laying under such conditions^69^. Studies under constant darkness (DD), which is predominate in natural bee nests or hives, generally show a lack of strong circadian rhythms in queen locomotor activity and egg-laying^69^. Queens tend to be active and lay eggs around the clock, a behavioral pattern thought to be linked to their high reproductive state and supported by the continuous, arrhythmic performance of many in-hive tasks, including queen care, by worker bees^70^.

Our finding that queen short-stops are uniformly distributed throughout the 24-hour day (Fig. 7) in hives maintained under DD conditions aligns well with previously reported findings^69,70^, even models designed to capture spatio-temporal patterns in pedestrian movement did not prove time to be usefull for queen localization^71^. It is important to note that our examination of temporal aspects in this particular analysis was specifically focused on the daily distribution of these short-stop events, rather than a comprehensive investigation into all potential temporal elements of the queen’s general locomotion or other behaviors. We view the absence of a discernible circadian pattern in the short-stop behavior as an indication that our DD conditions were sufficiently stable to avoid imposing external rhythmic cues on the behavior of the queens. Consequently, while the failure of simple random walk models in our study highlights significant uncaptured complexities in queen movement, the observed arrhythmicity in her short-stop behavior implies that this particular temporal factor is not the primary reason for the model inadequacies. Other elements, such as responses to local hive conditions, internal motivations beyond a daily clock, or more intricate search strategies, are more likely to explain the complex, non-random aspects of her movement patterns that our simple models failed to replicate.

### Queen and development of the hive

As discussed previously, strategies of movement have to balance between explorative and exploitative behavior; therefore, they could also be directly or indirectly reflected in the apparent power-law distribution between locations of short-stops as that is a subset of her queen’s movement likely tied to specific behavioral modes. We found that across all hives, distances between the short-stops followed a power-law distribution. If the travel between short-stop locations was the motivation for the queen’s movement, the power-law would make it analogous to a Lévy flight strategy—a type of random movement that can maximize the efficiency of uninformed searching for sparse patches of resources^72^. In two out of three hives, the values of the fitted Lévy exponent were close to 2, which is the optimal value for searching^37^, while the fit for the third hive differed, particularly at the start of the tail of the distribution. An extensive body of work was developed for researching evolutionary explanations of the origin of such behavioral patterns specifically in foraging, but this optimization strategy has been shown to be a more general solution in animal behavior^18,73,74^, even extending far beyond the animal kingdom, for example to the T-cells of the immune system^75^.

The events where the queen stops are related to multiple specific behaviors or interactions that cause the queen to stop briefly (such as cell inspection without oviposition, feeding, antennal contacts, or other interaction with workers around), but that also makes them a condition necessary for oviposition. Because the movement of the honeybee queen is a precursor to the formation of the spatial structure of the brood nest, this finding might be relevant to the modeling of the emergence and sustaining of the brood nest. As such models often assume the motion to be some kind of an exponentially governed random walk^16^, they might not be able to capture properties of the polynomially governed egglaying-site visiting. Although short-stops, as defined in our study, can include behaviors other than oviposition, this is unlikely to invalidate our conclusion. This reasoning is based on^51^, demonstrating that oviposition is the prevailing behavior, constituting a significant majority of similarly identified “candidate egglaying” events.

### Limitations of the study

Our study provides new insight into the movements of honeybee queens over a significant period of time, shedding light on their behavior and some of the internal dynamics of the colony. Observing a larger sample of queens would strengthen our findings, show more about the inter-colony variability and make the findings more generalizable. The detailed and extended observation protocol employed for each queen, while yielding rich datasets, is logistically and technically demanding, currently making large-scale replication across many more individuals a significant undertaking. Nevertheless, the consistency of certain core findings across the observed queens is notable, such as the minimal variation in short-term movement statistics across functional areas and the power-law distribution characterizing distances between potential egg-laying events, which suggests fundamental underlying movement strategies. Moreover, given the investigated hypothesis of a simple random walk behavior, which should be ergodic, we believe that the temporal extent partially makes up for a lower number of subjects. A potential limitation is the finite space within our two-frame observation hives, considering queens can lay hundreds to thousands of eggs daily at their peak^76^. However, several factors likely mitigated severe space constraints in this study. Observations were conducted in August-September, post-peak egg-laying season for Central Europe, when daily output is naturally reduced^69,77^. Crucially, our 22-day observation period corresponds to a worker brood cycle (approx. 21 days), meaning cells were continually emptied and prepared as brood emerged, ensuring dynamic space regeneration. This is consistent with our finding that the queen’s short-stop activity remained uniformly distributed throughout the 24-hour day (Fig. 7), a pattern less likely if she faced progressively severe cell shortages. While the observation hive environments are inherently more constrained, the post-peak season and stable short-stop activity suggest that a critical lack of egg-laying space was unlikely to have been a dominant factor systematically altering the investigated fundamental queen movement characteristics.

#### Technical context of the study

The field of animal tracking is advancing rapidly, with the use of versatile, AI-driven software and markerless methods becoming increasingly prevalent^78^. Such methods can be used to monitor entire honeybee colonies within hives, for example^48^. These markerless techniques offer significant advantages, including the elimination of physical tags and the capacity for multi-individual tracking. However, these current markerless systems require substantial computing power and can not ensure error-free re-identification of individuals. The specific demands of this study require precise, long-term tracking of a single individuum in the dense, frequently occluded hive environment. Another technology increasingly used in bee research is Radio-Frequency Identification (RFID), which excels at monitoring foraging activity by recording individuals passing fixed points, such as hive entrances^79^. However, detailed analyses of intra-hive movement ecology, which are central to our study, require more than presence/passage data. Achieving trajectories with RFID would necessitate an impractically dense array of readers.

Consequently, we employed the WhyComb marker-based system, which is specifically adapted for robust performance in cluttered hive conditions^49^. This approach provided certainty in individual tracking with minimal computational load for identification and, crucially, delivered the high-precision positional data essential for our detailed movement analyses. Other wireless technologies such as GPS or UWB that are used for larger spatial scales require receivers that are too large for in-hive usage^80^. These technologies also do not provide visual feedback so other interest points such as egglayings cannot be observed. Our system also benefited from the use of relatively inexpensive and adaptable camera hardware. WhyComb was chosen as the most suitable method to achieve the specific objectives of robust, long-term, high-precision tracking of a single queen for in-depth movement characterization.

#### Future work

Our study focused solely on the queen’s movement patterns, assessing whether simple, random walk-based models accurately describe her behavior. While our study covered a 22-day period, a study covering the entire season could provide deeper insights. It is expected that the queen’s movement will be affected by factors such as weather, time of year, resource availability, and overall colony fitness. There are, of course, more questions connected to the queen’s behavior in general, which go beyond the scope of this study. Case-study observations on one animal of queen’s sleeping, interaction, and similar behaviors have recently been presented^51^, putting together observations on different levels of a complex swarm behavior. The complexity of the entire colony makes studying individual behavior challenging due to the many hidden factors and interactions present.

## Conclusion

Our study sheds light on the spatial preferences and locomotive behavior of honeybee queens within their hives. While we expected to see large differences in the quality of the queen’s movement in different functional areas of the hive, we could only detect them in behavior patterns observed over a long time period. We did not find such a pronounced pattern in the short-term statistical properties of their walk. Thus, we postulate the presence of a certain behavioral persistence or environmental interactions that act in longer horizons beyond her immediate decision-making and cause an illusion of habits. Standard simple models of movement based on random walks have proven unable to replicate characteristics of the queens’ behavior, which is consistent with the presence of more complex repetitive patterns. We believe that our analysis of the basic properties of the queens’ movement will prove useful for future developments of models of the eusocial dynamics and hive development.

## Supplementary Information

Supplementary Information.

## Data Availability

The data collected and analyzed during the current study are available at 10.5281/zenodo.15682344. The code for the data analysis performed in our study is available at 10.5281/zenodo.15683480.
