# Supplementary material for: On the movement of the honeybee queen in the hive
Source: Sci Rep. 2025 Jul 1;15:20708. doi: 10.1038/s41598-025-07093-4 (PMC12217729; doi:10.1038/s41598-025-07093-4)
Supplement: Supplementary file 1 — Supplementary Information. [file 41598_2025_7093_MOESM1_ESM.pdf]

On the Movement of the Honeybee Queen in the Hive
Supplementary material

Jan Blaha, Martin Stefanec*, Jiří Janota, Daniel Nicolas Hofstadler,
Tomáš Rouček, Jiří Ulrich, Laurenz Fedotoff, George Broughton,
Tomáš Vintr, Farshad Arvin, Thomas Schmickl and Tomáš Krajník

Contents

Supplementary Methods 1: Dataset Collection and Processing 3

Supplementary Methods 2: Cleaning data 7

Supplementary Methods 3: Tracklets 8

Supplementary Methods 4: Activity Segmentation 9

Supplementary Methods 5: Modes of Diffusivity 11

Supplementary Methods 6: Estimating Fractal Dimension 12

Supplementary Methods 7: Changepoint Detection 13

Supplementary Methods 8: Fitting Random Walk Models 14

Supplementary Methods 9: Considered Models 16

Supplementary Methods 10: Simulation of Random Walk Models 18

Supplementary Methods 11: Moran's I 19

Supplementary Results 1: Complete Fitting Results 21

Supplementary Results 2: Estimates of the Fractal Dimension 24

Supplementary Methods 1: Dataset Collection and Processing

Honeybee Queen Detection System

The data are collected by stationary robotic units arranged around *observation hives.* Each observation hive contains two two-sided combs above each other and is enclosed by two glass panes from both sides. The glass is placed on both sides a few centimeters from the combs so the bees can move and act freely. The observation hive is connected to the outside environment by ⌀ 6 cm tube, freely accessible by bees all the time. For the experiment purposes, each comb side is numbered starting from 0 and going counter-clockwise from the right bottom when looking at the sideboard with the tube, Figure S1. The whole construction, consisting of stationary robots and the observation hive, is covered by a heavy black tarp. The tarp forms a tent and blocks all the outside light from accessing the hive. For illumination eight LED light bulbs 9 W@850 nm were used on each side of the hive pointing to the middle of the comb.

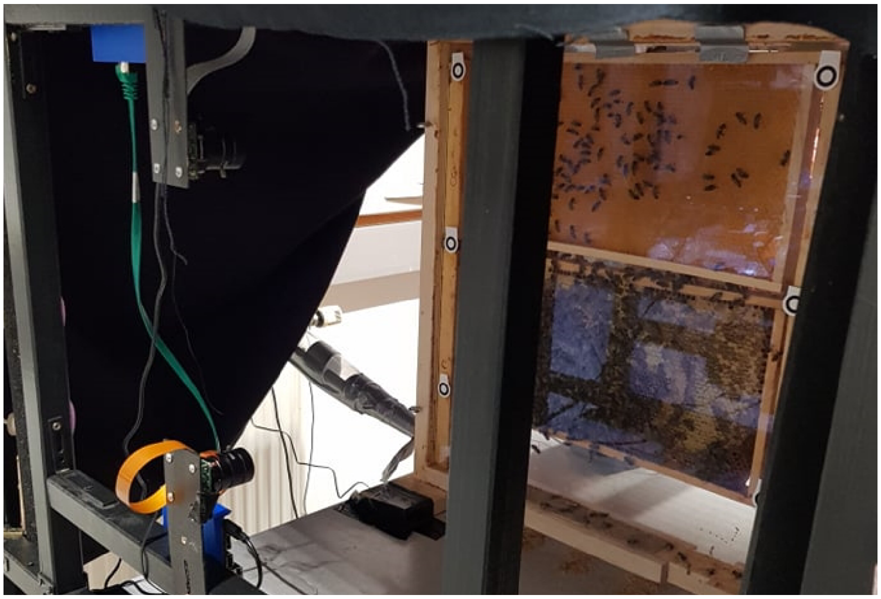

Figure S1: One side of the observation hive with two 4k cameras watching the combs. Large markers are visible on the sides as well as the tube that leads outside. Note the comb side numbering where the visible bottom comb is 0 and the upper is 1. Courtesy of[48], © 2022 IEEE.

Each side of each comb has a dedicated camera, ArduCam HQ-IMX477 4056x3040@10 Hz, with switchable IR filter (in our case always off) with CS2008ZM05A F/1.2 lens. Each camera is mounted 47 cm from the comb plane giving 1,720 px per cell or 63 px/mm^2 as shown in Figure S1.

On edges of glass panes are fiducial markers[49] with a ⌀ 17.88 mm that serve for getting an automatic camera alignment when the camera is accidentally moved during an event of cleaning the glasses or maintenance. Every camera is processed on its own NVIDIA Jetson Nano 2 GB computer, and data is stored on a master computer connected to each Jetson via ethernet. Due to hardware limitations, the images were captured by the system at 10 Hz.

To acquire a honeybee queen position in a plane of comb side, we use a small fiducial marker[49] of ⌀ 3 mm in diameter attached to her thorax. The marker comprises two concentric circles of known parameters and highly resembles the traditional markers used in the beekeeping industry. The marker is localised by the WhyComb system[48], an improved version of WhyCon[70] developed to overcome temporary partial occlusions. The detections are streamed to the central master computer, which coordinates the data recording and stores it in long-term storage. The master computer autonomously decides what data is going to be stored based on the visibility of the queen, reducing the amount of redundant records.

All involved computers run the operating system provided by the vendor, which is based on Ubuntu 18.04. The image acquisition and the queen detection are done within the Robot Operating System (ROS)[71] in

the version Melodic. Within this framework, individual software components can communicate in a unified abstract manner.

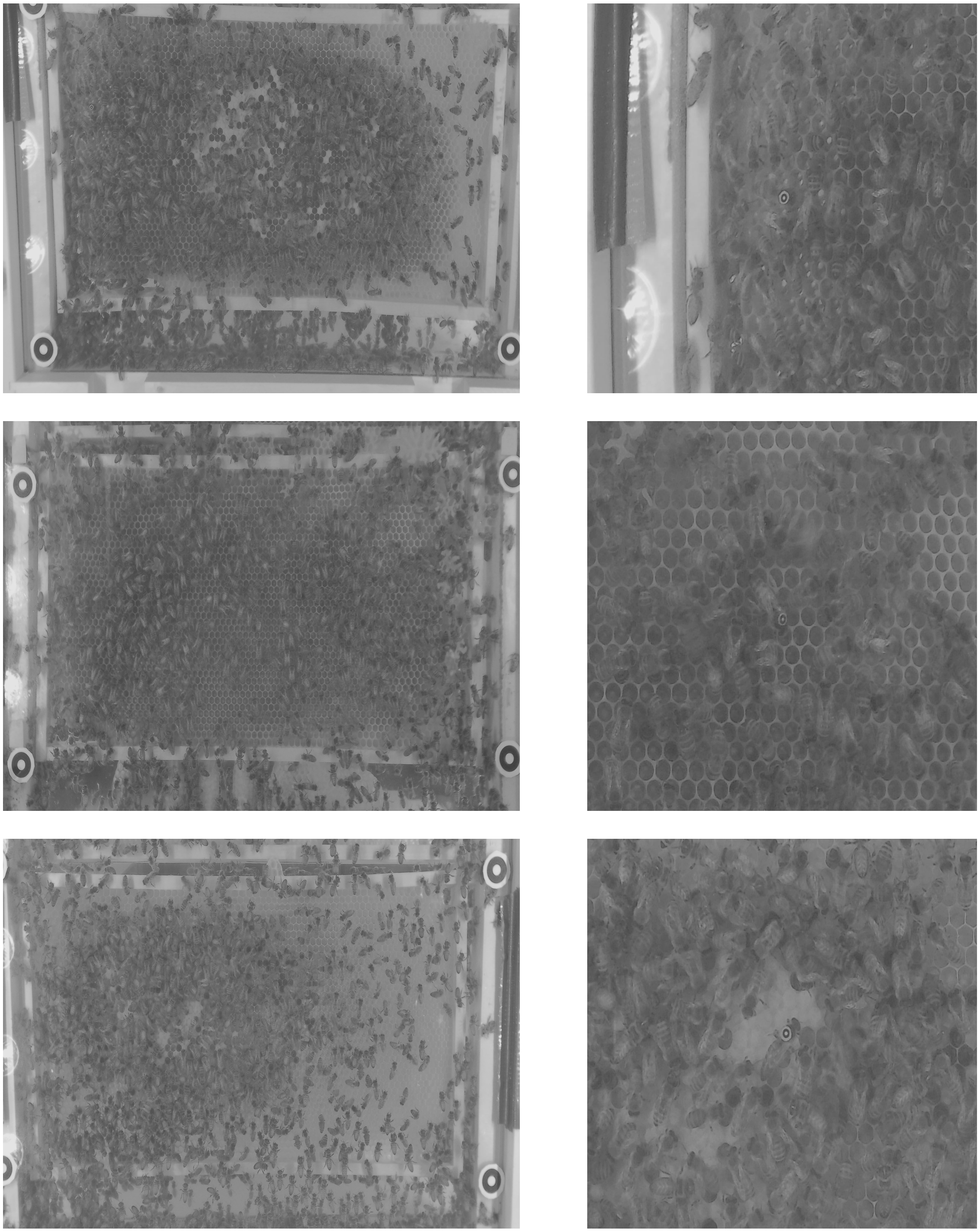

Figure S2: Examples of raw image data collected, no processing has been applied except undistortion. In rows, we show examples from individual hives 0, 1 and 2, respectively. The left column contains full images of one comb, and the right column shows recorded crops of $1{,}000 \times 1{,}000\,\mathrm{px}$ centered around the detected queen.

Published Data

The data was recorded between August 25, 2022 and September 15, 2022, covering in total 528 hours of recording. All data was recorded in one-hour-long blocks. The public dataset of raw honeybee queens detections from all observation hives is provided in CSV files with the structure described in Table S1. Derived from one-hour-long nature of original data, each CSV file is related to a specific hour and observation hive. The whole dataset is given in a compressed ZIP file (882 MB) of a structure shown in Figure S3.

In each CSV file, every row contains a unique index, UNIX timestamp in nanoseconds, identification from which camera the detection was recorded, and coordinates of the queen's position on the plane of the comb side given in meters. Next to that, it contains columns "whycomb_state" and "detection_confidence". An integer in "whycomb_state" is a value of a counter that is incremented by one each time WhyComb uses a convolution seeded on the previous position and reset to 0 when detection is done by the original WhyCon algorithm. More details can be found in the original publication[48]. As the reliability of WhyCon is very high, this information can be utilised for additional filtering of the false positives from the data. In the column "detection_confidence", we give the response from a neural network trained to recognize true and false positives. This number lies between 0 and 1 and represents the confidence of the neural network that it is a good detection (a true positive). For more details refer to Supplementary Methods 2.

Not all data have been properly recorded because of system downtime or hardware and software errors. Table S2 presents a list of hours with full downtime in tracking. The actual uptime of queen detections is shown in Figure S4 as a sequence of percentages of time the queen has been seen within 10-minute-long windows.

Names	Formats	Unit	Meaning
idx	int	-	Number of entry in csv
stamp	int	[ns]	Unix time of gathered data
frame_id	string	-	Camera id (hive and comb numbers)
x	float	[m]	X position of the queen
y	float	[m]	Y position of the queen
whycomb_state	int	-	Refer to text, range $[0,\infty)$
detection_confidence	float	-	Refer to text, range [0,1]

Table S1: Data structure of the published CSVs.

```
queen_september_2022_clean
├── 2022-08-25-00-00-01
│   └── ⋮
├── 2022-MM-DD-HH-MM-SS
│   ├── hive_0
│   │   └── detections_hive_0_with_pred.csv
│   ├── hive_1
│   │   └── detections_hive_1_with_pred.csv
│   └── hive_2
│       └── detections_hive_2_with_pred.csv
├── ⋮
└── 2022-09-15-23-00-02
    └── ⋮
```

Figure S3: The file structure of the provided ZIP file. Data are organized in the first level into folders containing data from individual hours, with the folder name being the timestamp in the format "YYYY-MM-DD-HH-MM-SS". For each hour, data are given as CSV files separately for each hive.

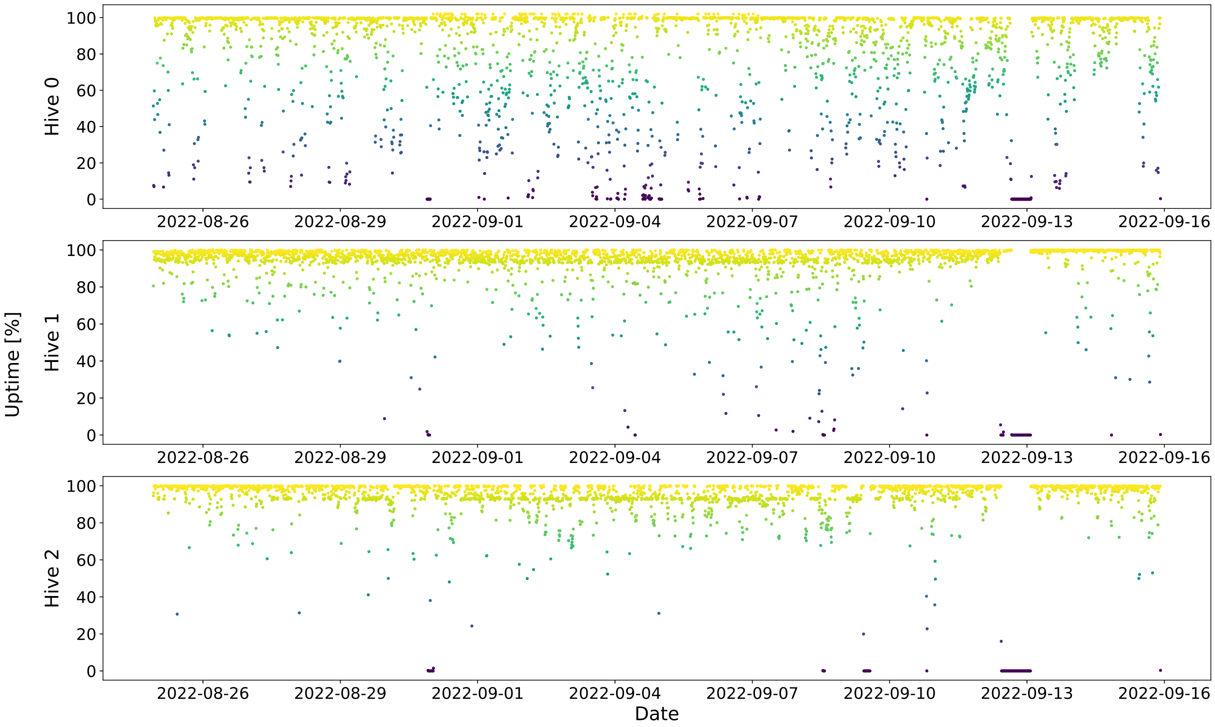

Figure S4: Recorded raw queen positions for each hive. Each dot represents how much percentage-wise has been recorded from the 10-minute window. Note the downtime on all hives at the end of August 31, 2022 and September 12, 2022.

Missing from	Missing to	Missing hours
2022-08-31-00-00-02		1
2022-09-12-18-00-01	2022-09-13-03-00-02	10

Table S2: List of downtimes spanning more than an hour. The provided CSVs are empty for the given times.

Supplementary Methods 2: Cleaning data

The collected data were in the form of noisy hour-long trajectories. We applied the following methods to filter out false positive detections using the original image data and the context of the trajectory. First, we employed a convolutional neural network (CNN) to classify true and false detections. We experimentally designed a compact 5-layer model (around 180k parameters) that classified images of size 256 × 256 px cropped around the detection of the honeybee queen (see Figure S5) into two classes—true and false detections. The CNN was trained on a manually annotated dataset that consisted of approximately 20 000 images. Due to the simplicity of the task, the trained model achieved 99.87 % precision and 99.43 % recall on validation data. Measurements that were classified as false detections were discarded.

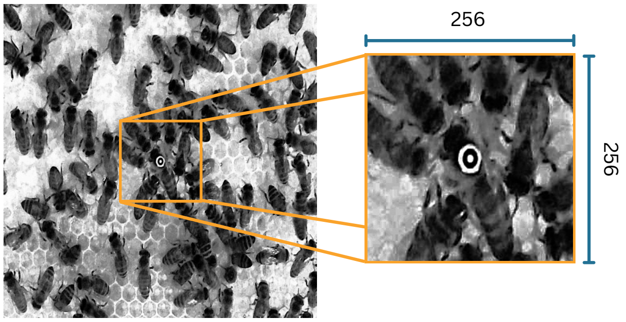

Figure S5: The convolutional neural network classified images of size 256 × 256 px cropped around the detection of the honeybee queen into two categories to filter false positive detections from true positive (example image). Note that the images have been adjusted for better readability by histogram equalization.

We considered the trajectories in the upper and bottom honeybee combs separately. When the queen was on a border between the combs, the trajectory usually contained measurements from cameras mounted on both honeybee combs. In that case, the trajectory was assigned to the honeybee comb from which the majority of the detections originated, and due to misalignments between the coordinate frames of the combs, the measurements from the other comb's camera were discarded from the trajectory.

Subsequently, we defined a set of rules based on queen kinematic constraints to eliminate the remaining false positive honeybee queen detections. The rules included filtering out steps with large velocities and significant jumps in the trajectory.

Finally, a Kalman smoother was employed for additional noise filtration and honeybee queen speed estimation. We defined the state vector of the honeybee queen as $(x, y, \dot{x}, \dot{y})^T$, where x and y are coordinates on the honeybee comb in meters, and $\dot{x}$ and $\dot{y}$ are their first derivatives, queen's speed. The particular setting of the parameters we used goes as

$$
\begin{aligned}
F &= \begin{pmatrix} 1 & 0 & \Delta t & 0 \\ 0 & 1 & 0 & \Delta t \\ 0 & 0 & 1 & 0 \\ 0 & 0 & 0 & 1 \end{pmatrix}, & H &= \begin{pmatrix} 1 & 0 & 0 & 0 \\ 0 & 1 & 0 & 0 \end{pmatrix}, \\
Q &= \begin{pmatrix} 10^{-4} & 0 & 0 & 0 \\ 0 & 10^{-4} & 0 & 0 \\ 0 & 0 & 10^{-2} & 0 \\ 0 & 0 & 0 & 10^{-2} \end{pmatrix}, & R &= \begin{pmatrix} 10^{-2} & 0 \\ 0 & 10^{-2} \end{pmatrix},
\end{aligned}
\tag{S1}
$$

where F is the state-transition matrix, H is the observation matrix, Q is the process covariance matrix and R is the covariance matrix of measurement noise.

Supplementary Methods 3: Tracklets

The cleaned data were split into continuous parts of the queen's trajectory ("tracklets") based on a set of predefined rules. The proposed rules were chosen heuristically based on experimentation and qualitative assessment of the achieved results. In a continuous sequence of observations of position $(o_i)_{i=1}^{n}$, where $t(o_i)$ denotes the time of measurement and $\overline{v}(i)$ is the average velocity in a window centered around ten consecutive measurements, the sequence was split between observations $i-1$ and i if and only if at least one of theses situations emerged:

1. the time gap $t(o_i) - t(o_{i-1}) > 15\,\mathrm{s}$ (temporal loss of tracking),
2. the time gap $t(o_i) - t(o_{i-1}) > 3\,\mathrm{s}$ and $\overline{v}(i) > 0.1\,\mathrm{cm/s}$ (temporal loss of tracking),
3. the distance $||o_i - o_{i-1}|| > 2\,\mathrm{cm}$ (spatial loss of tracking),
4. the moving average distances in centered window of size three $\mathrm{avg}_{(j,k)=(i-1,i-2),\cdots,(i+1,i)} ||o_j - o_k|| > 1\,\mathrm{cm}$ (spatial loss of tracking),
5. the distance $||o_i - o_{i-1}|| > 0.5\,\mathrm{cm}$ and $\overline{v}(i) < 0.1\,\mathrm{cm/s}$ (spatio-temporal loss of tracking),
6. a rolling variant of the generalized variance[72] - the determinant of covariance matrix in a window centered around thirty consecutive positions $|cov\{(o_j)_{j=i-15}^{i+14}\}| > 0.4\,\mathrm{cm}^2$ (uncertainty in detection),

The distribution of lengths of generated tracklets and the total coverage in individual hours can be seen in Fig. S6. Note that our ability to track the queen in Hive 1 significantly differed from other two hives.

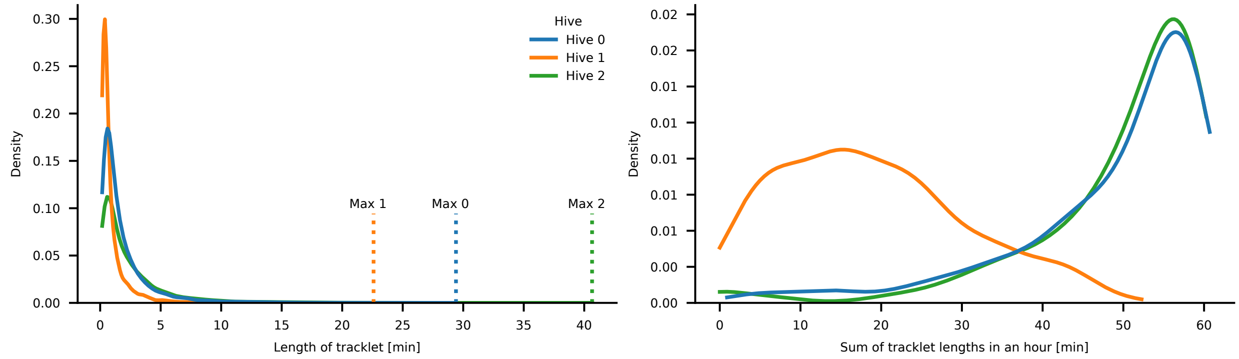

Figure S6: The left panel shows lengths of extracted tracklets per hive including maximal values. The right panel shows achieved coverage by clean tracklets in individual hours.

Supplementary Methods 4: Activity Segmentation

We designed a velocity-based classifier to identify the stopping and motion parts of the trajectories. For every hive, we manually annotated tracklets collected over one hour into the stopping and motion classes. Exploiting the estimated velocity of the honeybee queen from Kalman smoother, we estimate the density distribution of the speeds for both the motion and stopping phases. These distributions serve as a baseline for the activity segmentation.

During inference, we first calculate for each point of the trajectory with the corresponding velocity v the probability of being part of the stopping phase using Equation (S2), where $d_{\text{stopping}}(v)$ denotes the density of the speed in the stopping parts and $d_{\text{motion}}(v)$ denotes the density of the speed in motion parts. For individual measurement of the speed, we get the distribution, which can be seen in Figure S7.

$$P(v) = \frac{d_{\text{stopping}}(v)}{d_{\text{stopping}}(v) + d_{\text{motion}}(v)} \tag{S2}$$

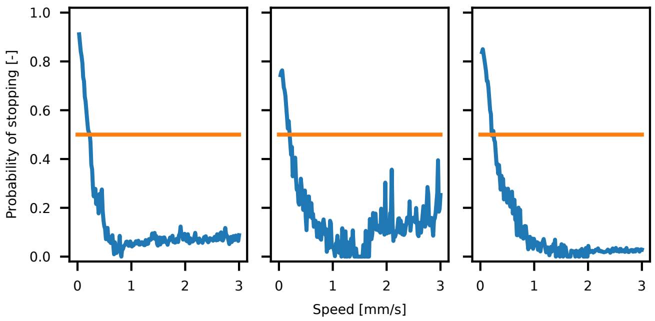

Figure S7: The probability of stopping, as defined in Eq. (S2), for speeds between zero and 3 mm/s for Hive 0, Hive 1, and Hive 2, respectively.

Our goal was to segment the recorded data into continuous stopping and motion phases. The probability of the individual point being part of the stopping phase $P(v)$ is not independent of the neighbouring measurements; so, we first smoothed the point probability estimates using a median filter with a time window of 7.5 s and a mean filter with a time window of 5 s to add contextual information. Then we thresholded the resulting stopping-phase probabilities using hive-specific thresholds T_1 (see Tab. S3), determined in exploratory analysis. After this classification of individual measurements into stopping and motion classes, we segmented the trajectory into segments of the same class.

Threshold type	Hive 0	Hive 1	Hive 2
T_1	0.60	0.50	0.55
T_2	0.80	0.70	0.78

Table S3: Thresholds on the probability of stopping which were used for the honeybee queen motion classification. Values were tuned manually with differences between hives caused mainly by the quality of data in each hive.

Following the expected biological reasons for the queen's stopping phase, we further categorised the stopping segments into short-stops (a work at a specific place) and long-stops (a rest). We identify the segment as a long-stop when one of two conditions holds

1. either the duration of the standing segment was longer than 120 s, or
2. the duration of the segment was longer than 30 s and the mean probability of stopping in the segment was greater than the experimentally determined hive-specific threshold T_2 (see Table S3).

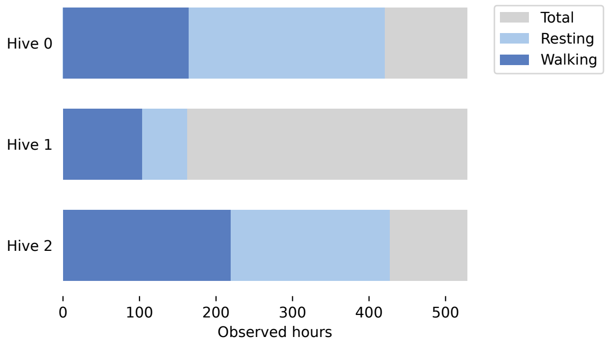

Figure S8: Proportions of resting and walking behavior for individual queens in our data together with proportions of covered time.

In other cases, the segment was classified as short-stop. The short-stop segments and motion segments were both processed together as data on the walking behavior of the queens. The long-stops were treated as proper resting behavior. The resulting proportions of recorded resting and walking times in individual hives classified using this method are shown in Fig. S8.

Supplementary Methods 5: Modes of Diffusivity

Diffusivity fundamentally determines the stochastic nature of propagation of the agent through the environment[51]. There are four basic diffusivity modes—subdiffusive, normal (Gaussian), superdiffusive, and ballistic. They are defined through mean-squared-displacement (MSD), which is the average distance from the start of the trajectory as a function of time.

For a process $(X_t)_{t>0}$, we define its diffusivity mode as

$$\langle X_t^2 \rangle \in \begin{cases} \mathcal{O}(t) & \text{subdiffusive} \\ \Theta(t) & \text{normal} \\ \Omega(t) \cap \mathcal{O}(t^2) & \text{superdiffusive} \\ \Theta(t^2) & \text{ballistic} \end{cases} . \tag{S3}$$

The normal mode—also referred to as "linear"—corresponds to the diffusivity of Brownian motion, and ballistic is the diffusivity of a walker on a straight line. From the normal mode, one can then define the terms "subdiffusivity" and "superdiffusivity", i.e., slower or faster than Brownian motion. Modes other than normal are also collectively called "anomalous diffusion".

Lévy walk is not a single model, so its MSD is not fully specified, and it is the superdiffusive mode of some Lévy walks that is intriguing. For the general Lévy walk $(L_t)_{t>0}$, with step lengths l distributed according to $p(l) \propto l^{-\mu}$ and where $\mu \in (1, 3]$ is then often called the Lévy exponent[50]. To avoid confusion, we note that in the literature, the heavy-tailed Lévy distribution is usually derived as a specific case of the stable laws, which are usually parametrized by $\alpha \in (0, 2]$, which also determines the commonly referenced Lévy exponent as $\mu = \alpha + 1$. The MSD of the walker then comes down to

$$\langle L_t^2 \rangle \propto \begin{cases} t^2 & 0 < \alpha < 1 \\ t^2/\ln t & \alpha = 1 \\ t^{3-\alpha} & 1 < \alpha < 2 \\ t \ln t & \alpha = 2 \\ t & \alpha > 2 \end{cases} . \tag{S4}$$

The derivation from stable laws limits our consideration for $\alpha \in (0, 2]$, but if one would start directly with the power-law distribution of steps, then α is not limited by 2 from the top. Following this the model exhibits ballistic diffusion for $0 < \alpha < 1$, super-diffusive sub-ballistic regime for $1 < \alpha < 2$, and standard diffusion for $\alpha > 2$[51].

When estimating the diffusivity, the definition of ensemble and time averages can, however, differ for processes governed by power-law-tailed step-distributions due to the weak-ergodicity-breaking (WEB). Fortunately, in our case, we only compare empirical observations, which we time average with simulations which are also time-averaged. Also, WEB manifests only as a change in the constant factor, which does not affect the diffusivity mode.

Supplementary Methods 6: Estimating Fractal Dimension

Trajectories of considered random walk processes create fractal patterns, which exhibit complexity greater than simple lines but not enough to constitute surfaces. A measure of such geometrical complexity of fractals is the fractal dimension, which is defined as an exponent of the scaling relationship between the size of the measuring device and the size of a given geometrical object. Formally, several definitions exist that all correspond to the same idea and even coincide in simple cases.

The most common definition, especially important for the simplicity of its application, is the so-called "box-counting" dimension. It is defined as

$$D = -\lim_{\epsilon \to 0} \frac{\log A}{\log \epsilon}, \tag{S5}$$

where A is the number of boxes of side ϵ in a regular grid that covers the geometrical object in question.

For our work, we decided to use the box-counting algorithm of[52], where we also included averaging over rotations and automatic selection of the range of suitable scales. Each tracklet was processed individually to compute its ϵ-area law and then average-pooled into ten randomly assigned groups. Slopes estimated for individual groups were then used for statistics and variance estimation.

The whole algorithm is in pseudocode described as Alg. 1.

Algorithm 1: Pseudocode of the algorithm for estimating fractal dimension

---

Input:
tracklets - a list of tracklets of one queen in one day,
n_rotations - number of rotations per each tracklet,
outer_box - the outer region for estimating area coverage,
δ_{cell_size} - step in the size of covering boxes (on log scale),
min_cell_size - minimal size of covering boxes,
reg_window_size - number of consecutive cell_sizes to estimate slope
Output:
D - estimate of the fractal dimension

$max\_cell\_size \leftarrow$ length_of_diagonal(box);
$N \leftarrow (max\_cell\_size - min\_cell\_size)/\delta_{cell\_size}$
for $i \leftarrow 0, \ldots, N$ **do**
  $cell\_size_i \leftarrow exp\{-(min\_cell\_size + i\delta_{cell\_size})\}$;
  foreach tracklet T in $tracklets$ **do**
    for $j \leftarrow 0, \ldots, n\_rotations$ **do**
      $T' \leftarrow$ rotate T by $j \frac{360}{n\_rotations}$ degrees;
      $a_{i,t,j} \leftarrow \#\{$boxes of edge $cell\_size_i$ in $outer\_box$ covering $T'\}$;
    $a_{i,t} \leftarrow \underset{j}{avg}\{a_{i,t,j}\}$
  $a_i \leftarrow \underset{t}{avg}\{a_{i,t}\}$;
for $i \leftarrow 0, \ldots, N - reg\_window\_size$ **do**
  $L_i \leftarrow$ fit linear model to $\left\{\log(cell\_size_{i+j}), \log(a_{i+j})\right\}_{j=0}^{reg\_window\_size}$;
  $e_i \leftarrow$ reconstruction error of L_i on training data;
  $s_i \leftarrow -$ slope of L_i;
$best\_window \leftarrow \underset{i}{argmin}\{e_i\}$;
$D \leftarrow s_{best\_window}$

Supplementary Methods 7: Changepoint Detection

With filtered data segmented into continuous tracklets, we needed to identify points of change of direction, approximating the trajectory with a polyline. We adopt a geometrical approach[53], where we compute the eigenvalues of a covariance matrix of data in some neighborhood and then inspect their ratio, which will be high, if the points are highly colinear and low, when there is a change of direction happening. Given a sequence of observations of in a tracklet $T = (\mathbf{o}_i)_{i=1}^n$, we compute

$$\begin{aligned} \forall i: \quad & O_i = \{\mathbf{o}_j \mid ||\mathbf{o}_k - \mathbf{o}_i||_2 \leq \epsilon \text{ for all } k = j, \ldots, i \text{ or } k = i, \ldots, j\} \\ & E_i = \{e_{i,1}, e_{i,2}\} = \lambda(cov(O_i)) \\ & r_i = \min E_i / \max E_i, \end{aligned} \tag{S6}$$

where O_i defines the trail-continuous ϵ-neighborhood of o_i, E_i is the set of two eigenvalues of the covariance matrix $cov(O_i)$ and finally r_i gives us the desired ratio. The changepoints are then defined as local maxima over r_i with added a condition for local non-maximal suppression (a minimal distance between indexes δ). We get indexes of changepoints as

$$I = \{i \mid r_{i-1} < r_i \wedge r_{i+1} < r_i \wedge \forall i, j \in I : |i - j| > \delta\}, \tag{S7}$$

where we identify local maxima and in case there are more within the distance δ we only keep the higher one. Changepoints then give the simplified trail $T' = (\mathbf{o}_j)_{j \in I}$.

In our implementation we used in particular values $\epsilon = 1\,\text{cm}$ and $\delta = 10$. We also noticed the need for smoothing of the data and experimentally gravitated towards sliding average in window of size 30 for the original data T, and further smoothing of the sequence $(r_i)_i$ with the window of size 20.

Supplementary Methods 8: Fitting Random Walk Models

In the literature previously investigating searching motion patterns in various animals, the main focus was mostly on the distributions of step lengths. Multiple methods were proposed to differentiate the power-laws from the originally expected exponential distributions. In the early studies, this was done by various versions of estimating the slope of a linear fit to log-transformed data, as that allowed for direct estimation of the power-law scaling exponent[73]. This, however, was later criticized extensively in[56,57], where many problems of these methods are discussed—the problem of quantization, missing data, bias towards certain estimates, and others. Ultimately, an approach based on fitting the candidate distributions by maximization of likelihood (MLE) was introduced with available methods of model comparison based on the Akaike information criterion (AIC). This was then adopted extensively in the literature, e.g., in[42,58,59]. Following the literature, we also adopt the approach based on the likelihood for fitting both the steps and turning angle. However, all of our models interact with the environment, so we have to rely on numerical models to compute their likelihood.

In unconstrained environments, the extremes of power-laws—unrealistic for real animal movement—are usually not treated specifically[56]. Because the comb is a highly confined area, we also consider generally constrained models, but for all our models, we have to deal with truncation of every single step individually, w.r.t the walker's position and orientation.

Specifically, fitting and validating power-law models is not completely straightforward, and there is still ongoing research on this topic. It is necessary to estimate the beginning of the empirical distribution's tail l_{min} first to determine the support of the power-law distribution, which effectively selects the relevant portion of the empirical data for fitting. This has been studied as a part of a method for general testing of the presence of power-laws in empirical data by[60,61]. In the case of truncated power-law distributions, one also needs to estimate the upper-truncation parameter l_{max}.

Formalization of the Fitting Process

We formalize our data as a set of simplified tracklets $\{T_j\}_j^N$, where one simplified tracklet is a sequence of changepoints $T_j = \left(\mathbf{t}_i\right)_{i=0}^n$, $\mathbf{t}_i = (t_x, t_y)$; see Fig. S9 for illustration. For fitting the turning angles distribution (AD), we will need to compute the change of direction ϕ, which we get from three consecutive changepoints $(\mathbf{t}_0, \mathbf{t}_1, \mathbf{t}_2)$ as

$$\phi = \begin{cases} \phi' & |\phi'| < \pi \\ -2\,\pi\,\mathrm{sign}(\phi') - \phi' & |\phi'| >= \pi \end{cases} \tag{S8}$$

$$\text{where} \quad \phi' = \mathrm{atan2}(d_{1,y}, d_{1,x}) - \mathrm{atan2}(d_{0,y}, d_{0,x})$$
$$\mathbf{d}_i = \mathbf{t}_{i+1} - \mathbf{t}_i$$

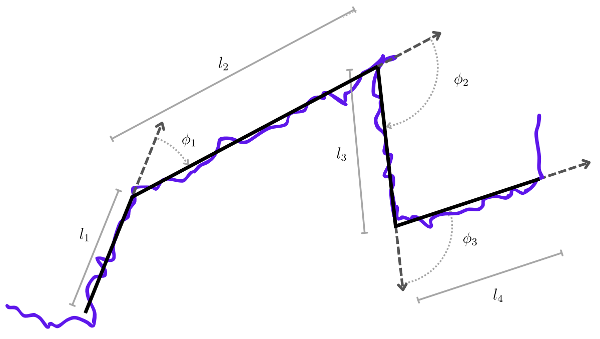

Figure S9: Diagram of polyline approximation of a tracklet with changepoints and turning angles.

Restriction to Comb Area

Fitting the distribution of the steps to observations $l_i = ||\mathbf{d}_i||$, $i = 1, \ldots, (n-1) \cdot N$, gets more difficult because the size of the area of the honeybee comb truncates it. For convenience, we denote $L_{\mathbf{t},\phi}$ as the distance from position $\mathbf{t}$ to the border of area A in the direction of ϕ. When referring to specific position and orientation at index i in data, we use $L_{\mathbf{t},\phi}(i)$. In all cases of step-length distributions, we need to truncate the distribution to accommodate for the constraints of A, i.e., the distribution with support $[a, \infty]$ has to become $[a, L_{\mathbf{t},\phi}]$. We denote this restriction of the distribution of steps S to the area A as $S|_{L_{\mathbf{t},\phi}}$. Because in likelihood computation, we deal only with probability density functions (PDF) and real observations that cannot fall outside this interval, we get the truncated version simply by renormalizing. This can be done by dividing the PDF by the value of the corresponding cumulative density function (CDF) at $L_{\mathbf{t},\phi}$.

Finding the Tail of the Step Distribution (Fitting l_{min})

As we care only about the tail of the actual distribution, we need to estimate l_{min}, where the tail starts. To select l_{min}, we use the approach of[60] where one would fit the distribution for some possible values of l_{min} and select the one where the theoretical distribution best matches the empirical one on $[l_{min}, \infty)$. The comparison between fitted distribution $S(\hat{\boldsymbol{\theta}})$ and the empirical data can be done using the Kolmogorov-Smirnov distance, where

$$KS = \max_{l \in X} |S_{CDF}(l) - O_{CDF}(l)| \tag{S9}$$

with $X = \{l \,|\, l > l_{min}\}$ being the restriction of data to the interval $[l_{min}, \infty)$, O_{CDF} being the empirical distribution function of the sample (ECDF) and S_{CDF} being the CDF of the distribution S. A specific in our case is that because of truncation to the comb area, each sample is actually drawn from a differently truncated distribution S, so we do not have access to its true CDF. We solve this by substituting the empirical mixture over observed truncations

$$\hat{S}_{CDF} = \frac{1}{|X|} \sum_i \left(S|_{L_{\mathbf{t},\phi}(i)}\right)_{CDF}. \tag{S10}$$

In our implementation, we chose potential values of l_{min} as 100 uniform quantiles of our empirical data.

Fitting Parameters of Step Distributions

The fitting of a distribution S with parameters $\boldsymbol{\theta}$ itself is done by maximizing the log-likelihood as $\hat{\boldsymbol{\theta}} = \arg\max \ell(\boldsymbol{\theta})$, where

$$\ell(\boldsymbol{\theta}) = \sum_{i,\ l_i > l_{min}} \log p_{S|_{L_{\mathbf{t},\phi}(i)}}(l_i; \boldsymbol{\theta}). \tag{S11}$$

A special case that needs commentary is the estimation of the truncation parameter of the Truncated Pareto distribution l_{max} (for details, see Supplementary Methods 9). The MLE would be to set it to the largest seen observation, which would not matter for exponential distributions so much as the likelihood of large values is not large, but for Pareto this likelihood is not negligible. To lessen the impact of this on the estimated parameters, we estimate it as $l_{max} = 1.3 \cdot \max_i \{l_i\}$ for observations l_i, to allow for larger unseen values.

Fitting Parameters of Angles Distributions

For the distributions of angles, we can use standard MLE estimates $\hat{\boldsymbol{\theta}} = \arg\max \ell(\boldsymbol{\theta})$. To compute them, we used numerical approximations by[54,55] to compute the estimates.

Bootstrapping Estimates

To get a better estimate of the parameters and also the corresponding errors, we use bootstrapping[74]. Once we fit l_{min} and we establish where the tail starts, we resample with replacement our data $L = \{l_i | l_i > l_{min}\}$ 1000 times getting $\{L'_j\}_{j=1,\ldots,1000}$. To these new samples, we then fit all distribution-specific parameters $\boldsymbol{\theta}$ getting $\{\hat{\boldsymbol{\theta}}_j\}$ and report mean $\hat{\boldsymbol{\theta}}_j$ and std $\hat{\boldsymbol{\theta}}_j$ for each parameter.

Supplementary Methods 9: Considered Models

This section provides details on our choice of candidate models to fit for the tail of steps distribution and angle distribution and how that relates to standard models of random walks. We also give an overview of the definitions of all these distributions.

- **Exponentially governed:** From its definition, Brownian walk is a random process starting at $\mathbf{0}$ with i.i.d. increments $\sim N(\mathbf{0}, \sigma * I)$, for scaling parameter σ. Deconstructing into the step-and-turn model, the distribution of its angles (AD) is $U(-\pi, \pi)$ and the distribution of steps (SD) is $Rayleigh_2(\sigma)$, which has a squared exponential tail. To model this class of walks, we fitted a squared exponential SD ("ExpSq") using a transformation to simple exponential, according to $l^2 \sim Exp(\lambda)$, where going back to the original parametrization $\lambda = 1/(2\sigma^2)$, or backward $\sigma = 1/\sqrt{2\lambda}$. Because the standard consideration for a step distribution in literature is the exponential distribution, we also include it in our candidate list ("Exp").

- **Polynomially governed:** For Lévy walk, the AD is $U(-\pi, \pi)$, and the SD is Lévy stable, with $Pareto(l_{min}, \mu)$ tail ("Par"). For the Truncated Lévy walk, the steps distribution can be truncated in two ways, so we consider both options. First is a fixed cut-off resulting in $TPareto(l_{min}, l_{max}, \mu)$ tail ("TPar"). Second is an exponential cutoff with tail $l \propto l^{-\mu}e^{-\nu l}$ on $[l_{min}, \infty]$ ("ExpTPar").

- **Correlated angles:** Considering the models of correlated random walks, the angles are distributed as $vonMisses(\rho, \kappa)$ instead of the uniform distribution. This distribution gives, on average, small changes in direction and, therefore, introduces some directional persistence even to the exponentially governed walks. It is standard to assume $\rho = 0$, but we wanted to allow for this free parameter as the queen could have a tendency to some circular motion, preferring one direction of circulation.

Details for individual distributions of steps are distilled in Tab. S4, where we split the PDF $p(l) = Cf(l)$ into the normalization constant C and shape term $f(l)$. We have to consider two constraints in our models—the lower bound given by the parameter l_{min} denoting the start of the tail of the distribution and the upper bound given by the constraints of the comb area. To accommodate for l_{min}, we use data transformation, discarding all $l_i < l_{min}$ and shifting $l'_i = l_i - l_{min} + a$, where a is the start of the support of our model, and we do this with the upper bound l_{tr} as well. To deal with the effect of the upper bound on the distribution, we renormalize it. We write $p|_A(l) = C_{tr}f(l) = Cf(l)/F(l_{tr})$, where F is the CFD respective to p, which allows to split C_{tr} into standard normalization constant C and truncation dependent term $1/F(l_{tr})$. The exception is "TPar", where the truncation cannot be split into C and $F(l_{tr})$, so we give full C_{tr}.

The models for the distribution of angles are given in the same fashion in Tab. S5.

Distribution	Shape term $f(l)$	Normalization constant C	Renormalization constant $F(l_{tr})$	Support	Params
Exp	$e^{-\lambda l}$	λ	$1 - e^{-\lambda l_{tr}}$	$[0, l_{tr}]$	λ
ExpSq	$e^{-l^2/(2\sigma)}$	-	-	$[0, l_{tr}]$	σ
Par	$l^{-\mu}$	$\mu - 1$	$1 - \frac{1}{l_{tr}}^{\mu-1}$	$[1, l_{tr}]$	μ
TPar	$l^{-\mu}$	$(\mu-1)(1-l_{max}^{-(\mu-1)})^{-1}$	$(\mu-1)(1-\eta^{-(\mu-1)})^{-1}$ *	$[1, \eta]$	μ, l_{max}
ExpTPar	$l^{-\mu}e^{-\nu l}$	$\nu^{1-\mu} \cdot \Gamma(1-\mu, \nu)^{-1}$	$\nu^{1-\mu} \cdot (\Gamma(1-\mu, \nu) - \Gamma(1-\mu, \nu l_{tr}))^{-1}$	$[1, l_{tr}]$	μ, ν

Table S4: Details of individual step distributions. For the purpose of decluttering terms of "TPar" distribution, we use an auxiliary symbol $\eta = \min(l_{max}, l_{tr})$.

Up until now, we only concerned ourselves with the tail of the distribution as that dictates most of the macro-level properties of the walk we care about. However, in order to simulate a walker given an appropriate

Distribution	Shape term $f(\alpha)$	C^{-1}	Parameters
Uniform	-	2π	
vonMisses	$e^{\kappa \cos{(\alpha - \rho)}}$	$2\pi I_0(\kappa)$	ρ, κ

Table S5: Details of individual angle distributions.

tail distribution, we also needed a model of the empirical distribution of steps on $[0, l_{min}]$. To approach the full step-laws present in the common random walk models, we did not use a uniform distribution. We aimed for a trivial model approaching 0 from the right side, so we fitted a half-normal distribution truncated to $[0, l_{min}]$ with the location set on l_{min} and a free scale parameter s.

Supplementary Methods 10: Simulation of Random Walk Models

To sample trajectories of the fitted random walks, we make use of the step-and-turn formulation, which allows us to sample steps and angle changes independently and then integrate them to get the full trajectory. In our experiments, we tried to match the characteristics of real observations, so for each empirical tracklet, we sampled several samples of the fitted model of the same length and uniformly random initial position in the comb area. To implement the behavior on the borders of the honeybee comb in line with our model definitions in Supplementary Methods 9, we rejected all sampled lengths that would lead to her ending up outside of the comb.

The step lengths are sampled according to the law:

$$l \sim \begin{cases} \alpha \; p_{tN(l_{min},s)} & 0 < l < l_{min} \\ (1-\alpha) \; p|_A(l) & l < \min(l_{tr}, l_{max}) \end{cases}, \tag{S12}$$

where $\alpha = q_{emp}(l_{min})$ is the quantile of the empirical data corresponding to the mass of the distribution on $[0, l_{min}]$ and tN is the normal distribution truncated to $[0, l_{min}]$. This defines a mixture that can be sampled hierarchically by first selecting the component using the alternative distribution with parameter α.

Supplementary Methods 11: Moran's I

Moran's I is one of the possible measures of spatial autocorrelation of observations on some fixed structure, usually described as a graph. We use a generalization to vector measurements[62], which is for set of vectors $\{\mathbf{x}_i\}$ indexed by $i \in J$, an index of location, defined as

$$I = \frac{n}{\sum_{i,j \in J} w_{i,j}} \frac{\sum_{i,j \in J} w_{i,j} \langle \mathbf{z}_i | \mathbf{z}_j \rangle}{\sum_{i \in J} \langle \mathbf{z}_i | \mathbf{z}_i \rangle}, \tag{S13}$$

where $\mathbf{z}_i = \mathbf{x}_i - \overline{\mathbf{x}}$ are centralized observations and $W = w_{i,j}$ is the neighborhood function/matrix. The W provides weights to quantify the proximity of two elements and can effectively encode any particular graph arrangement. Often, $w_{i,j}$ will be one for immediate neighbors and zero otherwise, but various alternatives are possible, like weighting based on geographical distance.

Moran's I allows us to quantify the amount of orderliness and test for it. The standard null hypothesis H_0 used is that there is no spatial arrangement of the observed measurements $\{\mathbf{x}_i\}$. This formulation makes it possible to approximate samples from the H_0 hypothesis by randomly rearranging the data between the locations J and computing the resulting PDF of $I|H_0$ (a non-parametric permutation-based estimate). This gives the expected value and standard deviation of $I|H_0$, which is used for computing the p-value of the actually observed I. The sampled distribution under H_0 and the actual measurement of the original data I give the so-called Moran's plot.

To fully define and apply Moran's I to our situation, we need to specify the weight matrix W. Our data has the form of a grid since we are dealing with mean vectors of direction grouped using a discretization of the comb space (we used 2 cm regular grid), but if no observations were made in a specific cell, its contents are undefined. We define our graph for I computation only over cells that do have a defined estimate of direction, and we allow nonzero weights only between cells that are close enough. We weight all pairs in by their Euclidean distance, so given a function c mapping positions $i \in J$ to their grid coordinates, the weights are defined as

$$w_{i,j} = \begin{cases} ||c(i) - c(j)||_2 & \text{if} \quad ||c(i) - c(j)||_1 < M \\ 0 & \text{otherwise} \end{cases}, \tag{S14}$$

which specifies W, but introduces a new parameter M of the maximal neighbourhood distance. We tested this parameter's effect and show the results for various sizes of neighborhoods in Fig. S10. In a combined plot, we give for each value of M a standard Moran's plot on the y-axis. Judging the results, we see that, in general, the results hold over the whole range of reasonable neighborhoods.

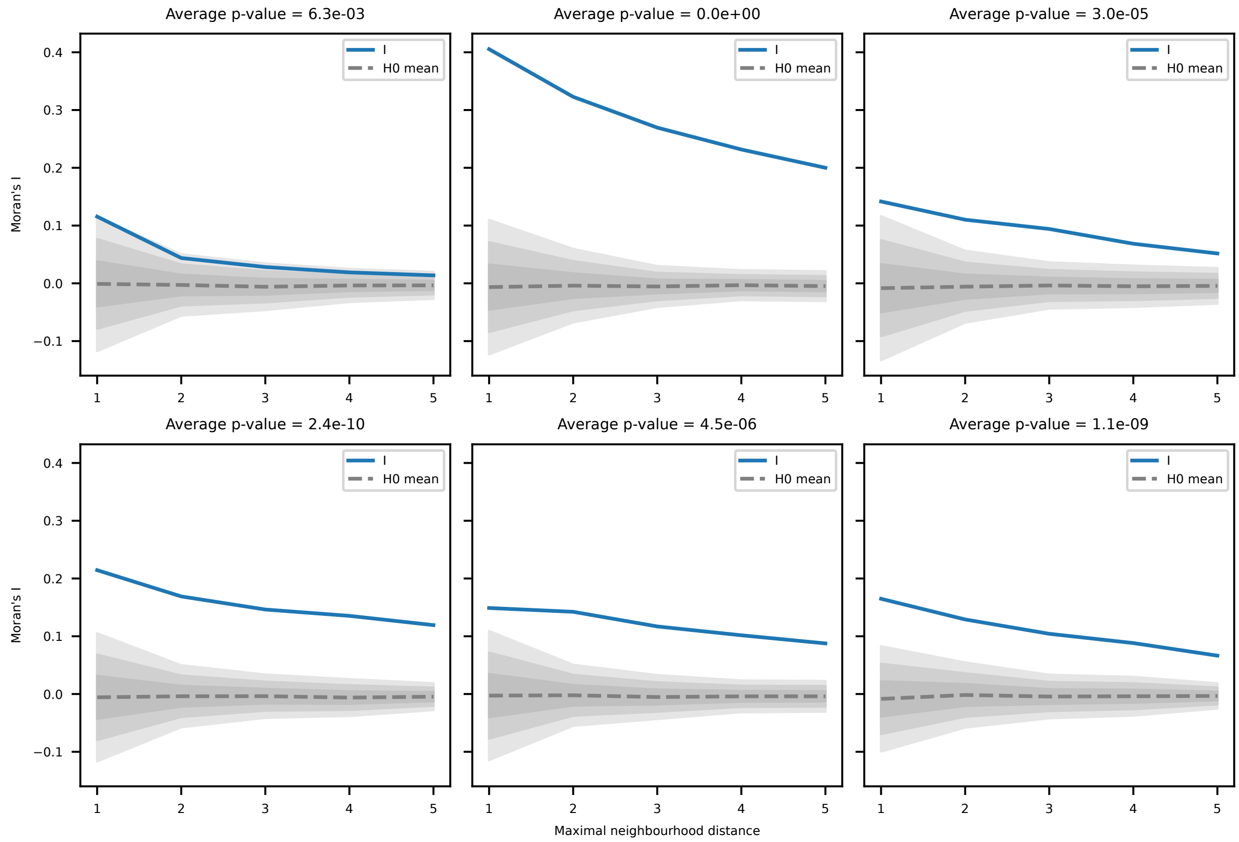

Figure S10: Moran plot for different values of the maximal Manhattan distance. Individual panels refer to individual combs (upper and lower row), with each column corresponding to one hive. The gray area shows the distribution of I_{H_0} (I under the H_0 hypothesis) with one, two, and three standard deviations mapped to color opacity. The blue line shows the actual I.

Supplementary Results 1: Complete Fitting Results

This supplementary section shows in completeness all the results of fitting steps and angle distribution models. Table S6 gives the "hyperparameters" common to all of the models, that is, the l_{min} denoting the start of the tail and s denoting the scale of truncated normal distribution used to simulate the rest of the body. Table S7 shows the parameters specific to individual distributions as defined in Supplementary Methods 9. All parameters that were bootstrapped—except for l_{min}—and are therefore given with the bootstrapped mean and standard deviation. Table S8 shows the Akaike information criteria (AIC) to provide a measure of goodness-of-fit, and Tab. S9 shows the relative Akaike weights for individual models. In bold, we denote the highest weight across the hive. The weights quantify the relative evidence towards individual models based on the achieved value of likelihood from the MLE estimation.

	Model	Parameter	Hive 0	Hive 1	Hive 2
Changepoints	Exp	l_{min}	2.07	1.52	1.97
		s	1.75 ± 0.13	0.91 ± 0.11	4.54 ± 2.26
	ExpSq	l_{min}	1.27	1.03	2.20
		s	0.98 ± 0.10	2.31 ± 2.19	10.55 ± 4.59
	Par	l_{min}	2.02	1.43	1.97
		s	1.66 ± 0.12	0.90 ± 0.13	4.48 ± 2.07
	TPar	l_{min}	2.02	1.40	2.07
		s	1.65 ± 0.12	0.91 ± 0.13	7.33 ± 3.97
	ExpTPar	l_{min}	2.21	1.40	1.99
		s	2.05 ± 0.17	0.91 ± 0.13	4.70 ± 2.27
Short Stops	Exp	l_{min}	0.28	0.01	0.41
		s	6.79 ± 0.15	0.10 ± 0.07	9.06 ± 0.22
	ExpSq	l_{min}	0.28	0.19	0.40
		s	6.78 ± 0.15	1.31 ± 2.00	8.96 ± 0.22
	Par	l_{min}	0.28	0.01	0.35
		s	6.79 ± 0.15	0.00 ± 0.00	7.90 ± 0.23
	TPar	l_{min}	0.28	0.01	0.35
		s	6.77 ± 0.15	0.00 ± 0.00	7.91 ± 0.23
	ExpTPar	l_{min}	0.28	0.01	0.35
		s	6.78 ± 0.15	0.00 ± 0.00	7.90 ± 0.22

Table S6: Fitted hyper-parameters of all considered models.

	Model	Parameter	Hive 0	Hive 1	Hive 2
Changepoints	Exp	λ	1.36 ± 0.07	1.55 ± 0.10	1.30 ± 0.04
	ExpSq	σ	1.16 ± 0.04	1.02 ± 0.04	1.00 ± 0.03
	Par	μ	2.98 ± 0.07	3.14 ± 0.10	2.90 ± 0.05
	TPar	μ	2.81 ± 0.10	2.64 ± 0.15	2.67 ± 0.07
		l_{max}	7.91 ± 0.52	4.64 ± 0.20	6.64 ± 0.27
	ExpTPar	μ	0.21 ± 0.32	0.01 ± 0.00	0.01 ± 0.00
		ν	1.29 ± 0.17	1.50 ± 0.08	1.29 ± 0.04
	VonMis	ρ	0.01 ± 0.05	0.01 ± 0.05	0.01 ± 0.03
		κ	0.69 ± 0.04	1.55 ± 0.10	0.72 ± 0.03
Short Stops	Exp	λ	0.59 ± 0.20	4.01 ± 0.69	0.44 ± 0.06
	ExpSq	σ	3.31 ± 0.72	0.77 ± 0.23	4.07 ± 0.64
	Par	μ	2.25 ± 0.36	6.27 ± 0.83	1.98 ± 0.12
	TPar	μ	2.14 ± 0.39	6.18 ± 0.84	1.97 ± 0.12
		l_{max}	10.14 ± 0.99	2.55 ± 0.64	23.58 ± 5.01
	ExpTPar	μ	2.20 ± 0.36	5.84 ± 1.45	1.87 ± 0.21
		ν	0.01 ± 0.04	0.32 ± 1.21	0.03 ± 0.06
	VonMis	ρ	-0.38 ± 0.55	-1.10 ± 2.21	0.46 ± 0.84
		κ	0.25 ± 0.10	0.34 ± 0.15	0.12 ± 0.06

Table S7: Fitted parameters of all considered models.

	Model	Hive 0	Hive 1	Hive 2	
Changepoints	Steps	Exp	490.53	240.73	1100.03
		ExpSq	554.50	250.26	1128.31
		Par	528.58	276.51	1223.64
		TPar	519.59	255.78	1170.73
		ExpTPar	492.07	242.57	1102.45
	Angles	VonMis	5925.04	1288.99	13265.65
		Unif	6296.57	1654.09	14166.36
Short Stops	Steps	Exp	113.39	18.12	482.10
		ExpSq	134.16	21.08	568.91
		Par	95.20	10.80	456.57
		TPar	96.09	12.62	458.28
		ExpTPar	97.20	12.79	458.57
	Angles	VonMis	652.45	230.16	1780.91
		Unif	650.61	227.90	1779.07

Table S8: AIC for all considered models.

	Model	Hive 0	Hive 1	Hive 2	
Changepoints	Steps	Exp	**0.68**	**0.71**	**0.77**
		ExpSq	0.00	0.01	0.00
		Par	0.00	0.00	0.00
		TPar	0.00	0.00	0.00
		ExpTPar	0.32	0.28	0.23
	Angles	VonMis	**1.00**	**1.00**	**1.00**
		Unif	0.00	0.00	0.00
Short Stops	Steps	Exp	0.00	0.01	0.00
		ExpSq	0.00	0.00	0.00
		Par	**0.50**	**0.55**	**0.56**
		TPar	0.32	0.22	0.24
		ExpTPar	0.18	0.20	0.21
	Angles	VonMis	0.29	0.24	0.28
		Unif	**0.71**	**0.76**	**0.72**

Table S9: Akaike relative weights for all considered models.

Supplementary Results 2: Estimates of the Fractal Dimension

Table S10 gives the estimates of the fractal dimension of the observed data, Tab. S11 gives the complete results of the estimates of fractal dimension D of the simulated random walk models.

Hive	Comb	Mean
Hive 0	Lower	1.111 ± 0.005
	Upper	1.076 ± 0.011
Hive 1	Lower	1.083 ± 0.008
	Upper	1.058 ± 0.002
Hive 2	Lower	1.125 ± 0.005
	Upper	1.072 ± 0.008

Table S10: Fractal dimension of the simulated models.

Model	Hive	D
Exp	Hive 0	1.089 ± 0.002
	Hive 1	1.003 ± 0.001
	Hive 2	1.098 ± 0.002
ExpSq	Hive 0	1.089 ± 0.002
	Hive 1	1.004 ± 0.001
	Hive 2	1.099 ± 0.003
ExpTPar	Hive 0	1.088 ± 0.001
	Hive 1	1.003 ± 0.001
	Hive 2	1.100 ± 0.002
Par	Hive 0	1.088 ± 0.002
	Hive 1	1.003 ± 0.001
	Hive 2	1.097 ± 0.002
TPar	Hive 0	1.085 ± 0.002
	Hive 1	1.001 ± 0.001
	Hive 2	1.093 ± 0.002

Table S11: Fractal dimension of the simulated models.

References

[70] Krajník, T. et al. A practical multirobot localization system. J. Intell. Rob. Syst. **76**, 539–562, DOI: 10.1007/s10846-014-0041-x (2014).

[71] Quigley, M. et al. Ros: An open-source robot operating system. In 2009 IEEE International Conference on Robotics and Automation (ICRA) Workshop on Open Source Robotics (2009).

[72] Wilks, S. S. Certain generalizations in the analysis of variance. Biometrika **24**, 471–494, DOI: 10.1093/biomet/24.3-4.471 (1932).

[73] Sims, D. W., Righton, D. & Pitchford, J. W. Minimizing errors in identifying Lévy flight behaviour of organisms. J. Anim. Ecol. **76**, 222–229, DOI: 10.1111/j.1365-2656.2006.01208.x (2007).

[74] Efron, B. Bootstrap methods: Another look at the jackknife. Ann. Stat. **7**, 1–26 (1979).
